# Cooperation and competition shape ecological resistance during periodic spatial disturbance of engineered bacteria

**DOI:** 10.1038/s41598-017-00588-9

**Published:** 2017-03-27

**Authors:** Cortney E. Wilson, Allison J. Lopatkin, Travis J. A. Craddock, William W. Driscoll, Omar Tonsi Eldakar, Jose V. Lopez, Robert P. Smith

**Affiliations:** 1 Department of Biological Sciences, Halmos College of Natural Sciences and Oceanography, Nova Southeastern University, 3301 College Ave, Fort Lauderdale, Florida 33314 USA; 2 Guy Harvey Oceanographic Center, Nova Southeastern University, 8000 North Ocean Dr, Dania Beach, Florida 33004 USA; 3 Department of Biomedical Engineering, Duke University, 101 Science Drive, Durham, North Carolina USA; 4 Clinical Systems Biology Group, Institute for Neuro-Immune Medicine, Nova Southeastern University, 3301 College Ave, Fort Lauderdale, Florida 33314 USA; 5 Department of Psychology & Neuroscience, College of Psychology, Nova Southeastern University, 3301 College Ave, Fort Lauderdale, Florida 33314 USA; 6 Department of Computer Science, College of Engineering and Computing, Nova Southeastern University, 3301 College Ave, Fort Lauderdale, Florida 33314 USA; 7 Department of Clinical Immunology, College of Osteopathic Medicine, Nova Southeastern University, 3301 College Ave, Fort Lauderdale, Florida 33314 USA; 8 Department of Ecology, Evolution, and Behavior, University of Minnesota, 100 Ecology, 1987 Upper Buford Circle, St. Paul, Minnesota 55108 USA

## Abstract

Cooperation is fundamental to the survival of many bacterial species. Previous studies have shown that spatial structure can both promote and suppress cooperation. Most environments where bacteria are found are periodically disturbed, which can affect the spatial structure of the population. Despite the important role that spatial disturbances play in maintaining ecological relationships, it remains unclear as to how periodic spatial disturbances affect bacteria dependent on cooperation for survival. Here, we use bacteria engineered with a strong Allee effect to investigate how the frequency of periodic spatial disturbances affects cooperation. We show that at intermediate frequencies of spatial disturbance, the ability of the bacterial population to cooperate is perturbed. A mathematical model demonstrates that periodic spatial disturbance leads to a tradeoff between accessing an autoinducer and accessing nutrients, which determines the ability of the bacteria to cooperate. Based on this relationship, we alter the ability of the bacteria to access an autoinducer. We show that increased access to an autoinducer can enhance cooperation, but can also reduce ecological resistance, defined as the ability of a population to resist changes due to disturbance. Our results may have implications in maintaining stability of microbial communities and in the treatment of infectious diseases.

## Introduction

Cooperation plays a vital role in many biological systems, including within bacterial populations^1^. Here, cooperation is critical in coordinating diverse behaviors^2^ including the colonization of the rhizosphere^3^, activation of virulence genes^4^, host colonization^5^, antibiotic resistance^6^, and the production of bioluminescence^7^.

Cooperation between bacteria can be regulated by the production and exchange of small diffusible molecules or peptides called autoinducers in a process called quorum sensing^8^. Cells sense and respond to the local autoinducer concentration, which often serves as a trigger for downstream gene expression. If the concentration of the autoinducer is too low, the cooperative behavior is not initiated. As the majority of the autoinducer is not retained by the bacterium that produces it, the concentration of the autoinducer detected by each bacterium can be largely dictated by the spatial distribution of bacteria^9^. Previous theoretical and experimental studies have demonstrated that, in undisturbed environments, spatial structure, or clustering, of bacteria can facilitate cooperation^10–14^. Conversely, in some instances, spatial structure has also been shown to undermine cooperation^15–17^. Furthermore, spatial structure can lead to reduced growth through increased competition^18, 19^, which may further reduce the fitness of cooperative microbes. Nevertheless, cooperation remains stable in nature, thus suggesting that mechanisms exist to balance these conflicting observations. These mechanisms have yet to be fully described.

The majority of previous studies that have examined how spatial structure influences cooperation have occurred in either undisturbed or continuously disturbed (well-mixed) environments. However, these studies predominantly investigated the stability of cooperation in the presence of non-cooperating ‘cheater’ cells^20^. Furthermore, they did not examine the dynamics of populations that rely on cooperation for survival, also known as a strong Allee effect^21^. Strong Allee effects have been observed in many systems including non-invasive^22^, invasive^23^ and reintroduced species^24^. In bacteria, strong Allee effects are observed when pathogenic bacteria infect a host^5^, during activation of virulence factors^25^, in the formation of antibiotic resistant biofilms^26^, and during cooperation of planktonic bacteria to resist an antibiotic^27^.

Disturbances are rarely an all or none occurrence as periodic disturbances play a critical role in microbial communities^28^. Environments where bacteria are naturally found, including soils^29^, marine environments^30^, and in hosts^31^, are subject to periodic disturbances^32^. These disturbances may cause reorganization of spatially structured organisms, including bacteria^33, 34^. Depending on the ability of the population to resist such changes (i.e., ecological resistance), these disturbances may influence the ability of a population to cooperate. Moreover, how disturbances affect microbial communities has been historically challenging to study given multiple confounding variables that can impact natural populations^28^. Thus, it currently remains unclear as to how periodic spatial disturbances affect bacterial populations dependent on cooperation for survival. Understanding how spatial structure driven by periodic disturbances affects cooperation is critical to understanding and manipulating cooperative bacteria, which may allow for unique approaches to reduce the growth of pathogenic bacteria^35^.

In this manuscript, we used engineered bacteria designed using the principles of synthetic biology. Engineered microbes have been previously used to unveil unique dynamics that are often challenging, if not impossible, to observe and quantify in their natural counterparts^36, 37^. These engineered systems serve as a middle ground between empirical studies, which may suffer from multiple confounding variables, and purely theoretical or mathematical studies, which may not necessarily operate in biologically feasible parameters spaces. Owing to the usefulness of engineered microbial systems, many theoretically predicted cooperative dynamics have been successfully observed in engineered microbial systems^38^, while new dynamics have also been described^39^. Our engineered bacteria use quorum sensing to regulate survival and growth. Along this line, cooperative dynamics regulated by quorum sensing have been previously studied in engineered bacteria^40^. For example, Pai *et al*., used an engineered *Escherichia coli* strain to demonstrate conditions under which quorum sensing is beneficial to a population^41^. Furthermore, Smith *et al*., used engineered bacteria that require the expression of a quorum sensing regulated gene to survive to demonstrate tradeoffs between cooperation and dispersal^42^. Finally, Zhang *et al*., used two engineered strains of *Bacillus subtilis* to examine tradeoffs between quorum sensing and the production of extracellular matrix during biofilm formation^43^.

## Results

### An experimental approach to study the effects of periodic spatial disturbance on cooperation

**Figure 1:**
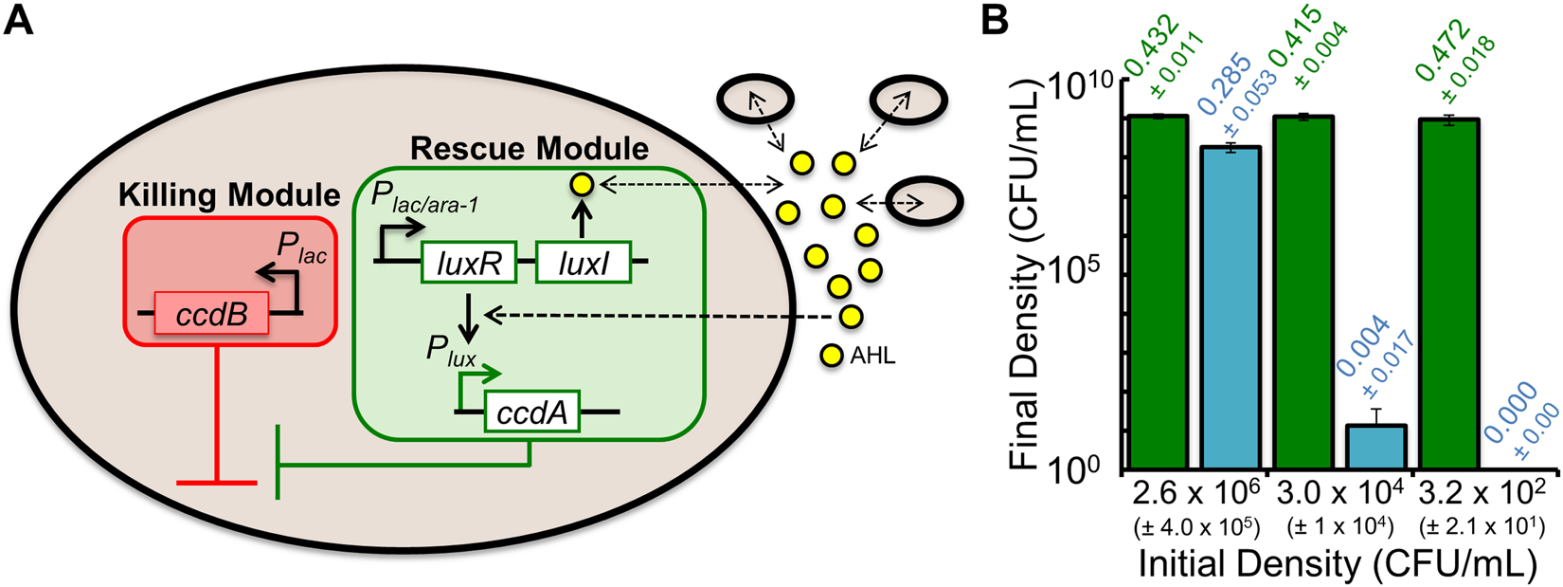
The engineered bacteria used in this study. (**A**) The gene circuit consists of two modules: a killing module (red shading) and a rescue module (green shading). The killing module contains an IPTG inducible (*P*_*lac*_ promoter) *ccdB* gene from the CcdA/CcdB toxin-antitoxin system. The rescue module contains an IPTG inducible (*P*_*lac*/*ara*-*1*_ promoter) *luxR*/*luxI* quorum-sensing (QS) system from *Vibrio fischeri* ^8^ and an AHL inducible (*P*_*lux*_ promoter) *ccdA* from the CcdA/CcdB toxin-antitoxin system. Induction with IPTG (1 mM) causes expression of *luxR*/*luxI* and *ccdB*. *ccdB* causes cell death^44^. However, *ccdA* can inhibit *ccdB* if *luxI* synthesizes a sufficient amount of AHL (yellow circles), which is dependent upon the initial density of bacteria in the population. (**B**) Final density of engineered bacteria (CFU/mL) in cultures incubated for 48 hours starting from different initial densities (see Methods for details) Green bars = no IPTG, circuit off. Blue bars = IPTG, circuit on. An OD_600_ of 0 indicates that no CFUs were detectable in the medium. Standard deviation (SD) from a minimum of three biological replicates.

To investigate how periodic spatial disturbance affects a population with a strong Allee effect, we used an engineered strain of *E*. *coli* that requires access to a shared acylhomoserine lactone (AHL) to grow and survive^42^ (Fig. 1A). The circuit is activated through the addition of isopropyl β-D-1-thiogalactopyronoside (IPTG) to the medium. Upon circuit activation, the bacteria express the CcdB toxin protein, which can kill the bacteria^44^. The addition of IPTG simultaneously drives expression of LuxI^8^, a protein that produces AHL. Once a sufficiently high concentration of AHL is reached, it activates the expression of the CcdA anti-toxin protein, which inhibits the CcdB toxin protein and allows population growth, and survival. If AHL does not reach a sufficiently high concentration, expression of *ccdA*, and thus cooperation, does not occur and the population goes extinct. Since AHL rescue only occurs at sufficiently high AHL concentrations, and therefore a sufficiently high cell density, the ability to successfully cooperate is measured using the minimal density of bacteria required for population growth (*P*_*CRIT*_). Enhanced cooperation corresponds to a lower value of *P*_*CRIT*_. Experimentally, we defined *P*_*CRIT*_ as the highest initial density of bacteria where the cell density (OD_600_) after 48 hours of growth is not statistically different than zero^42^. We confirmed that an OD_600_ value of zero at 48 hours represents, on average, an absence of colony forming units (CFU, Fig. 1B). This indicated that cooperation was not successful.

**Figure 2:**
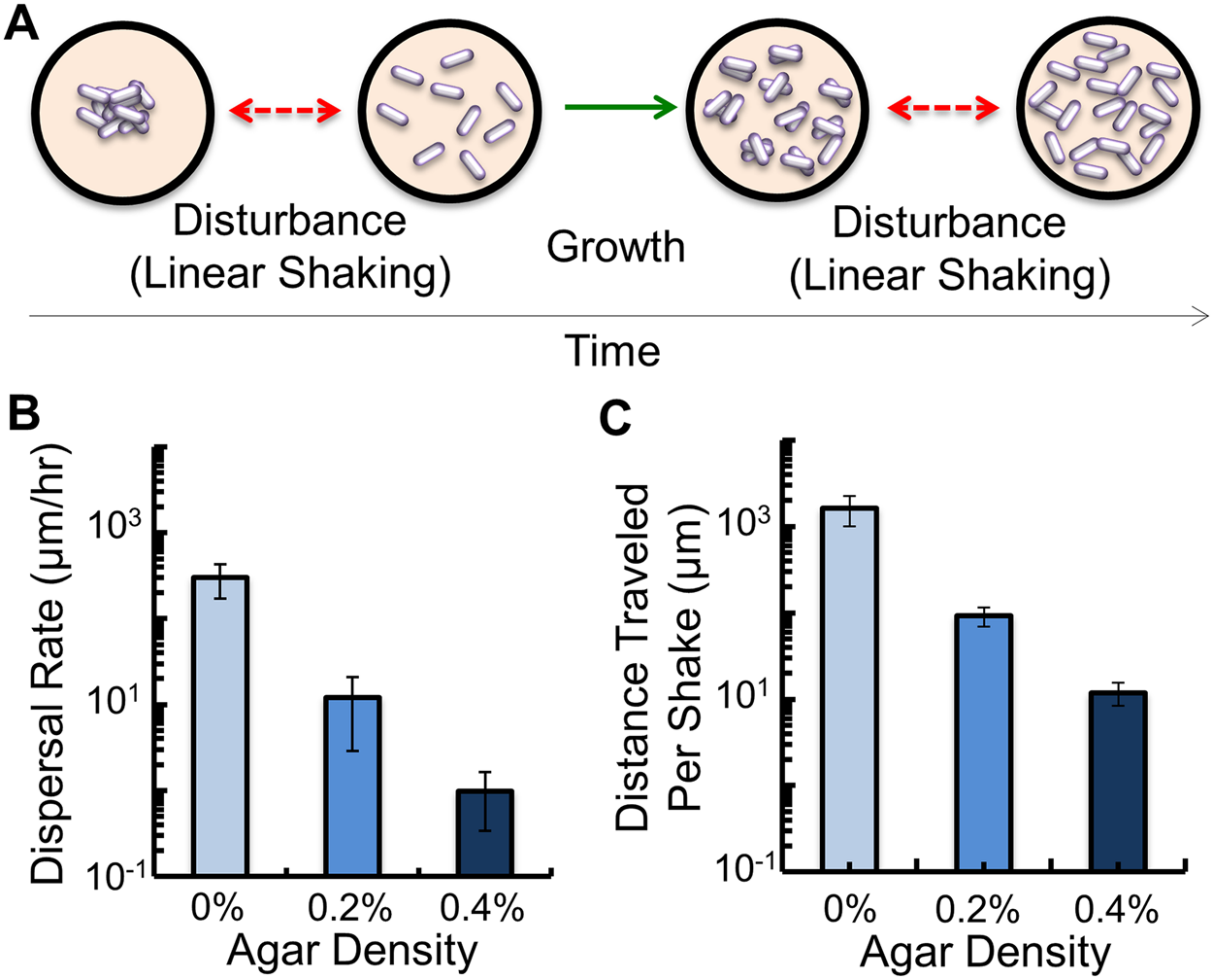
Quantifying the movement of *gfp*-expressing bacteria during linear shaking. (**A**) We periodically altered the spatial distribution of the bacterial population by shaking the microplate linearly. This dispersed the bacteria away from their positions where, after a period of time, their positions were disturbed again. (**B**) Average dispersal rate of bacteria in medium with different agar densities, and in the absence of shaking (see Methods). *p* ≤ 0.03 amongst all conditions (two-tailed t-test in panels (A,B)). SD from six biological replicates. (**C**) Average distance travelled by the bacteria after a single shake in the microplate reader (see Methods). For all comparisons, *p* ≤ 0.05. SD from a minimum of three biological replicates.

We grew our engineered bacteria in a 96-well microplate to determine the effect that periodic disturbance had on cooperation and survival (see Methods). Previous studies have demonstrated that changing the agar density in growth medium is an effective method to alter the spatial distribution of microbes^45^. To achieve this, we dissolved different amounts of agar in the medium (0%, 0.2% and 0.4%), which would serve to change the degree to which each spatial disturbance altered the distribution of bacteria. To simulate a spatial disturbance, we periodically shook the microplate in a microplate reader (Fig. 2A) using a linear pattern with a 0.1 mm radius. To confirm that this shaking pattern was sufficient to displace bacteria, we used a *gfp*-expressing strain of bacteria and measured how far the bacteria travelled in a linear cell culture chamber after an individual shake (Methods). We observed that increasing the agar density in the medium reduced the distance each individual shaking event dispersed the bacteria (Fig. 2B,C). We note that shaking the micoplate to cause a disturbance is analogous to a pulse type disturbance, which are observed in nature and are short term disturbances that are discrete in time^46^. In this study, we chose to use a generic and previously described classification of disturbance, defined as “any relatively discrete event in time that disrupts ecosystem, community, or population structure and changes resources, substrate availability, or the physical environment”^47, 48^. Analogous to shaking in the microplate reader, disturbances can be an abiotic physical force or process that results in a perturbation to an ecosystem or population^49^.

**Figure 3:**
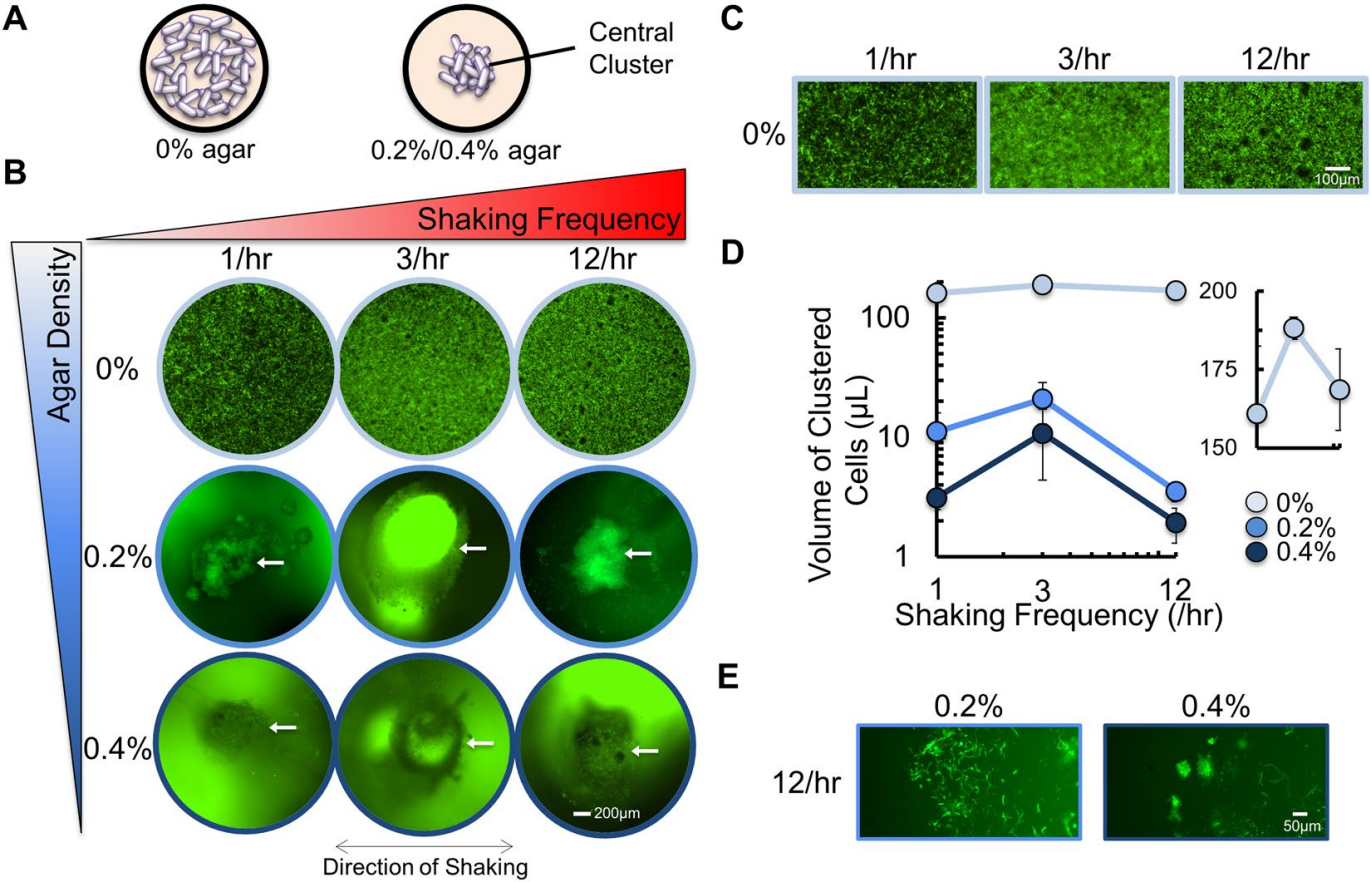
Periodic shaking of *gfp*-expressing bacteria in a microplate reader alters their spatial distribution. (**A**) When *gfp*-expressing bacteria were grown in medium with 0% agar, bacteria were spread amongst the well. When bacteria were grown in medium with 0.2% or 0.4% agar, the majority of bacteria were confined to a central cluster at the initial point of inoculation. (**B**) Representative images of *gfp*-expressing bacteria during periodic shaking of the microplate. The central cluster is indicated with a white arrow. For panels B, C and E, images taken at 24 hours. (**C**) Representative images of bacteria in 0% agar with increased magnification. (**D**) The volume of clustered *gfp*-expressing bacteria. In all agar densities, the volume of bacteria was highest at an intermediate shaking frequency (3/hr). Inset: Data expanded to show trend in medium with 0% agar. *p* *<* 0.048 when 1/hr and 12/hr are compared to 3/hr demonstrating significance in biphasic trend (one-tailed t-test). Volume calculated as described in Methods. SD from a minimum of three biological replicates. (**E**) Representative images of bacteria outside of the central cluster. Bacteria were scattered, less numerous and formed smaller colonies.

Next, we used fluorescent microscopy to observe how different frequencies of shaking would determine the spatial distribution of the bacteria (Fig. 3). To accomplish this, we inoculated a 100-fold dilution of an overnight culture of *gfp*-expressing bacteria into the center of a well in a 96- well plate that contained M9 medium. When initially inoculated into medium with 0% agar, bacteria appeared to spread uniformly throughout the medium (Fig. 3A), and did not exclusively settle to the bottom of the well. When inoculated into medium with 0.2% and 0.4% agar, the majority of the bacteria remained clustered around the initial point of inoculation (Fig. 3A), and were distributed throughout the height of the well. Here, the bacteria formed a shape that was reminiscent of a cylinder.

We then shook the microplate at different frequencies and examined the distribution of *gfp*-expressing bacteria at 24 and 48 hours. Each shaking frequency resulted in a unique change in the spatial distribution of bacteria (Fig. 3B–E). With 0% agar, low or high shaking frequency [e.g., 1 shake/hr (1/hr) or 12 shakes/hr (12/hr)] resulted in defined areas of bacterial growth, which we call bacterial clusters (Fig. 3B. Magnified areas of bacteria in 0% agar shown in Fig. 3C). At intermediate shaking frequency [e.g., 3 shakes/hr (3/hr)], bacterial growth appeared more evenly distributed throughout the medium, with few areas lacking bacterial growth. With 0.2% and 0.4% agar in the medium, the majority of the bacteria grew in the center of the well as a column, which we call the central cluster. The volume of the central cluster followed a biphasic trend with shaking frequency, where its size was maximized at a shaking frequency of 3/hr and the total size was reduced with increasing agar density (Fig. 3D). Outside of the central cluster, bacteria were unorganized or formed smaller, irregular clusters, were reduced in number and appeared to occur at different heights in the well (Fig. 3E).

### Access to nutrients and autoinducer determine population survival during periodic spatial disturbance

**Figure 4:**
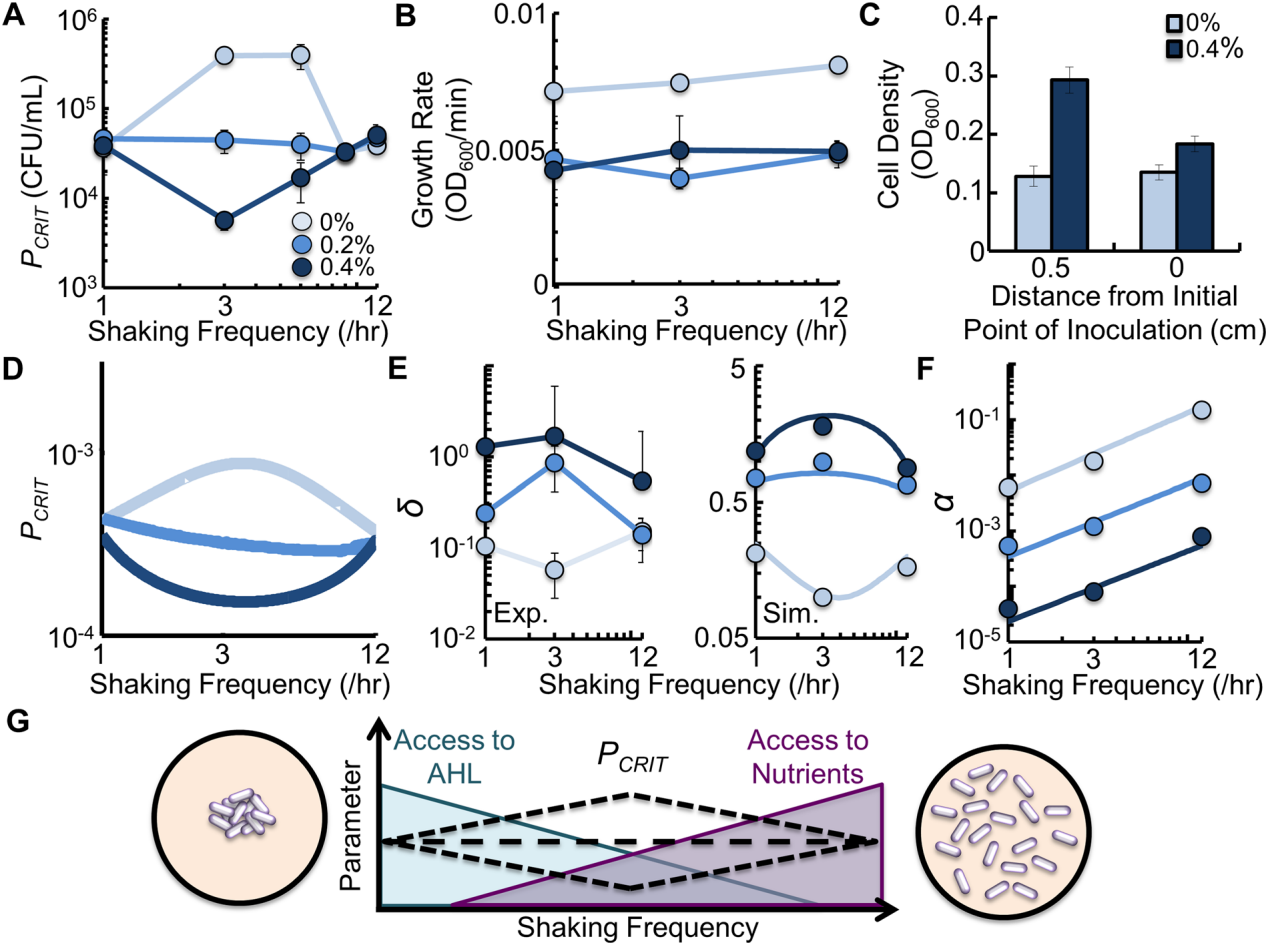
Access to AHL and access to nutrients determines *P*_*CRIT*_ during periodic spatial disturbance. (**A**) *P*_*CRIT*_ of engineered bacteria shaken at different frequencies and agar densities. At 1/hr, 9/hr and 12/hr, *P*_*CRIT*_ was identical in all agar densities (*p* ≥ 0.36). At 3/hr and 6/hr, *P*_*CRIT*_ decreased with increasing agar density (*p* ≤ 0.04). Raw data in Supplementary Fig. S1. *P*_*CRIT*_ as a function of *δ* in Supplementary Fig. S2. Two-tailed t-tests for *P*_*CRIT*_ in Supplementary Tables S1 and S2. For panels with experimental data, SD from a minimum of three biological replicates. (**B**) Growth rate of engineered bacteria at different frequencies and agar densities. Bacteria in medium with 0% agar had a higher growth rate than bacteria in 0.2% and 0.4% agar (*p* ≤ 0.002, two-tailed t-test). *p* = 0.53 when 0.2% and 0.4% agar are compared to each other. (**C**) OD_600_ reached by bacteria after growing for 16 hours in medium obtained from previous cultures with 0% and 0.4% agar. In both cases, the medium was obtained at 0 cm and 0.5 cm from the initial point of inoculation. In 0% agar, OD_600_ was the same after 16 hours (*p* = 0.574). In 0.4% agar, OD_600_ was higher in the sample taken 0.5 cm away from the initial point of inoculation (*p* = 0.004, Methods and Supplementary Results). (**D**) Simulation results demonstrating how *P*_*CRIT*_ is affected by shaking frequency (Equations (1–3), see Methods). (**E**) Values of *δ* from experimental data (left panel) and in our model (right panel, see Methods). For experimental data, *p* ≤ 0.05 for comparisons within and between different frequencies and agar densities except *p* = 0.27 when 1/hr and 12/hr are compared in medium with 0.4% agar (two-tailed t-test). (**F**) Values of *α* in our model that were qualitatively fit to Fig. 2C (see Supplementary Results). (**G**) Schematic of mechanism. With low shaking frequency, bacteria are clustered, increasing AHL access but reducing nutrients access through increased competition. With high shaking frequency, AHL access is reduced, but access to nutrient is increased through decreased competition. The non-linearity of these opposing trends cause changes in *P*_*CRIT*_ at intermediate shaking frequency.

Using the experimental approach described above, we inoculated a 10-fold dilution series of the engineered cooperative bacteria into the center of microplate wells that contained M9 medium, and shook the population at different frequencies. We observed that when the microplate was shaken at 1/hr, 9/hr and 12/hr, *P*_*CRIT*_ was equivalent regardless of agar density in the medium (Fig. 4A). At intermediate shaking frequency (3/hr and 6/hr), *P*_*CRIT*_ decreased with increasing agar density. Here, cooperation was enhanced with 0.4% agar and inhibited with 0% agar. Otherwise, with 0.2% agar, cooperation was not altered relative to a low or high shaking frequency. All values of *P*_*CRIT*_ and their corresponding *p*-values are in Supplementary Table S1. For a statistical comparison between *P*_*CRIT*_ values, see Supplementary Table S2.

Intermediate frequencies of disturbance are a key feature in maintaining ecological relationships, including the preservation of biodiversity^50^ and species co-existence^51^. In general, an intermediate frequency is one that lies between the undisturbed, or continuously disturbed extremes^52^. We sought to understand why intermediate shaking frequencies led to changes in *P*_*CRIT*_. We formulated a mathematical model consisting of three delayed differential equations that considers bacterial growth, production of AHL, and first-order AHL-mediated rescue (Equations (1–3) in Methods.) Model development and assumptions can be found in the Supplementary Results. Parameters are presented in Supplementary Table S3.

To capture the effect of shaking the microplate, we consider two populations: *P*_*c*_, which are the bacteria inside clusters (including the central cluster), and *P*_*o*_, which are the bacteria outside of clusters. For each shaking event, bacteria predominantly move from *P*_*c*_ to *P*_*o*_ at a rate, *α*, which is controlled by agar density and shaking frequency. It has been previously observed that bacteria that grow in clusters or colonies have reduced growth rates due to nutrient limitation through increased competition^18^. We confirmed that bacteria in *P*_*c*_ grow slower (Fig. 4B) and that this is due to nutrient limitation (Fig. 4C, Methods). Therefore, *P*_*c*_ and *P*_*o*_ grow at different rates, *μ*_*c*_ and *μ*_*o*_, respectively, and that *μ*_*c*_ < *μ*_*o*_.

Clustering has been shown to facilitate local AHL accumulation^53^. Therefore, to incorporate this spatial effect into our two-dimensional model, we assume that AHL production is proportional to bacterial clustering, *δ*. Here, *δ* is a function of the change in the volume of clustered bacteria normalized by the volume over which the bacterial population is spread. Thus, *δ* serves as a scaling factor for AHL production, which incorporates the benefit of clustering for increased AHL access and simplifies the influence of periodic shaking. Large values of *δ* correspond to a highly clustered environment. As bacteria become less clustered, a decrease in *δ* diminishes the feedback of AHL production on the entire population (see Methods for estimation of *δ*). Experimentally, we quantified *δ* by estimating the volume occupied by clustered *gfp*-expressing bacteria (see Methods, Equation (4)). We used these experimentally determined values to guide *δ* in our model.

Our model predicts that intermediate shaking frequencies result in larger changes in *P*_*CRIT*_, as compared to high or low shaking frequency (Fig. 4D). When bacteria are shaken infrequently (1/hr) *δ* is sufficiently large (Fig. 4E), which serves to enhance cooperation. However, since *α* is sufficiently low (Fig. 4F), the growth rate is slow due to reduced nutrients through increased competition. As such, most bacteria grow at *μ*_*c*_. In contrast, when bacteria are shaken frequently (12/hr), *δ*, and therefore AHL production, is low. However, high values of *α* allow more bacteria to disperse away from the central cluster to grow faster at *μ*_*o*_. Here, access to nutrients is increased, reducing competition, which increases the growth rate of the population. This compensates for decreased AHL production from *P*_*c*_ by generating more bacteria in *P*_*o*_, each of which is capable of AHL production. As such, cooperation is driven by two opposing factors: growth rate, which is determined by access to nutrients and competition, and access to AHL, which is determined by bacterial clustering. At sufficiently low or high shaking frequencies, changes in *δ* and growth rate (*μ*_*c*_, *μ*_*o*_) serve to balance each other, resulting in minor differences in *P*_*CRIT*_ across agar densities (Fig. 4G).

Nonlinear changes in *δ* at intermediate shaking frequencies (e.g., 3/hr) lead to larger changes in *P*_*CRIT*_. When few bacteria disperse relative to the increase in *δ*, as is observed in 0.4% agar, cooperation is enhanced and *P*_*CRIT*_ decreases. Here, a small, but sufficient population of bacteria is dispersed, and grow at *μ*_*o*_. These bacteria gain increased access to nutrients, which serves to increase AHL production, and thus enhance cooperation. Conversely, when *α* does not sufficiently increase to compensate for the decrease in *δ*, as is observed in 0% agar, cooperation is reduced and *P*_*CRIT*_ increases. Here, increased access to nutrients allows for additional growth at *μ*_*0*_. However, because dispersal is not sufficiently high, additional growth at *μ*_*0*_ cannot compensate for the reduction in bacterial clustering. This reduces cooperation. When *α* and *δ* are balanced, which occur in 0.2% agar, *P*_*CRIT*_ remains relatively unchanged relative to low or high shaking frequency. Overall, at an intermediate shaking frequency, cooperation can be either enhanced or reduced by periodic spatial disturbances due to an imbalance between *δ* and growth (*μ*_*c*_ or *μ*_*o*_). We note that the inclusion of nonlinear rescue via AHL does not qualitatively impact the results of our simulation (Supplementary Fig. S3). Furthermore, if *δ* decreases as a function of shaking frequency, our model cannot predict our experimental results (Supplementary Fig. S3). Finally, sensitivity analysis revealed that our simulation results are robust when the values of most of our parameters in Equations (1–3) (see Methods) are varied between 0.5X and 2.5X (Supplementary Fig. S4).

### Stabilizing autoinducer leads to tradeoffs between maintaining ecological resistance and enhancing cooperation

**Figure 5:**
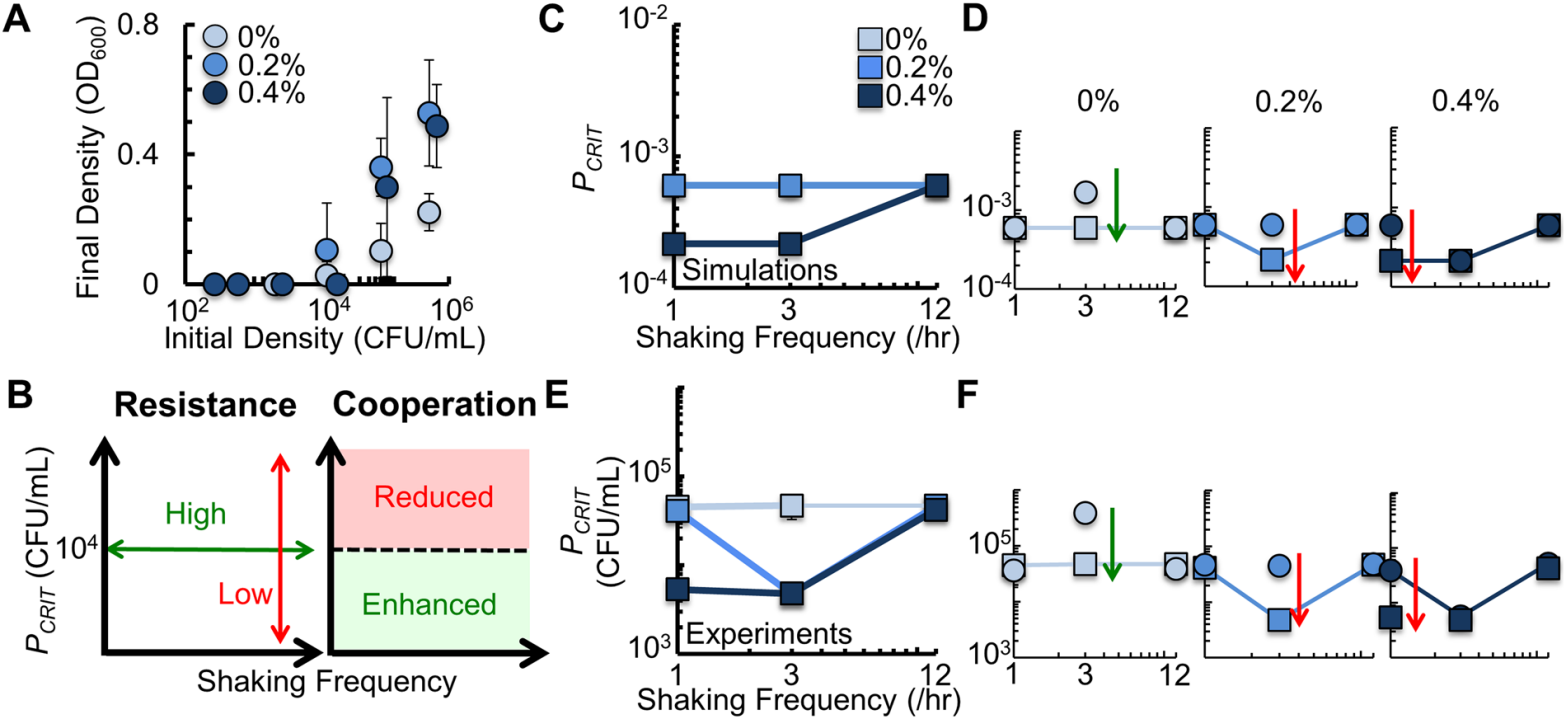
Increasing access to AHL perturbs the ability of bacteria to resist periodic spatial disturbance. (**A**) *P*_*CRIT*_ of engineered bacteria grown in different agar densities but in the absence of shaking. Average *P*_*CRIT*_ observed in all three agar densities was 4.84 × 10^4^ ± 1.91 × 10^4^ CFU/mL (*p* = 0.12, two-tailed t-test). Two-tailed t-tests for each *P*_*CRIT*_ in Supplementary Tables S1 and S2. SD from twelve measurements, consisting of at least three biological replicates per agar density. (**B**) Measuring resistance and cooperation. Left: In an undisturbed environment, *P*_*CRIT*_ was ~10^4^ CFU/mL. If the bacteria were resistant to disturbance, *P*_*CRIT*_ would remain at this density (green arrow). Changes in *P*_*CRIT*_ show a decrease in resistance (red arrow). Right: Cooperation is enhanced (green) or reduced (red) if *P*_*CRIT*_ is reduced or increased from ~10^4^ CFU/mL, respectively. (**C**) Simulation results (Equations (1–3)) showing *P*_*CRIT*_ with decreased *k*_*d*._ For simplicity, we show the three shaking frequencies measured experimentally. For a high-resolution simulation, consult Supplementary Fig. S5. (**D**) Individual simulations with decreased *k*_*d*_. Squares indicate simulation data performed with decreased *k*_*d*_. Circles indicate simulation data re-plotted from Fig. 4D. For panels (D,F), green arrows indicate increased resistance. Red arrows indicated reduced resistance. Direction of arrow indicates perturbation to *P*_*CRIT*_. (**E**) *P*_*CRIT*_ of engineered bacteria grown in medium with pH 7.0. Two-tailed t-tests for each *P*_*CRIT*_ in Supplementary Tables S1 and S2, including comparisons between *P*_*CRIT*_ in medium with pH 7.4 and pH 7.0. SD from a minimum of three biological replicates. (**F**) Individual experiments for each agar density. Squares indicate *P*_*CRIT*_ from bacteria grown in medium with pH 7.0. Circles are re-plotted from Fig. 4A. Raw data in Supplementary Fig. S5.

Ecological resistance is an important metric in ecological stability used to describe the amount of disturbance an ecosystem or community can withstand before being perturbed^54^. As microbial systems are a cornerstone of many ecosystems, understanding the principles that allow the maintenance of their ecological stability is critical in maintaining ecological relationships^28^. Often, resistance is measured by examining changes in community composition or function before and after a disturbance event, relative to an undisturbed control^55^. In line with this approach, resistance could be measured in our system as the ability of a given shaking frequency to change *P*_*CRIT*_. As a control condition, we used a *P*_*CRIT*_ on the order of magnitude of 10^4^ CFU/mL (4.84 × 10^4^ ± 1.91 × 10^4^ CFU/mL), which was observed when the bacteria were not shaken linearly (Fig. 5A). Along this line, bacteria were more resistant to periodic disturbance when shaking was applied at 1/hr, and 12/hr (Fig. 4A), as *P*_*CRIT*_ was not changed relative to the undisturbed population (*p* ≥ 0.12). Otherwise, resistance was reduced at an intermediate shaking frequency (3/hr) as *P*_*CRIT*_ was observed to change (increase and decrease, *p* < 0.001) relative to the undisturbed population (except 0.2% agar, *p* = 0.64).

Access to autoinducers, such as AHL, is a critical feature driving many cooperative behaviors in bacteria and is often determined by spatial distribution^56^. We sought to perturb AHL access to determine its affect on resistance (Fig. 5B, left) and cooperation (Fig. 5B, right). To capture increased access to AHL in our model, we reduced the AHL degradation term in Equation (1) (*k*_*d*_). Our simulations predict that at high shaking frequency, increasing AHL stability has a negligible influence on *P*_*CRIT*_ (Fig. 5C,D). If the bacteria are benefiting from increased access to nutrients (*μ*_*o*_) via large *α*, then decreasing *k*_*d*_ should result in minimal changes to cooperation. Here, resistance remains relatively unchanged. At 1/hr, decreasing *k*_*d*_ can decrease *P*_*CRIT*_, as is observed in 0.4% agar. In this case, a low *α* coupled with the benefits of a sufficiently large *δ* can reduce *P*_*CRIT*_. Here, reduced resistance promotes enhanced cooperation.

At 3/hr, increasing AHL access can have diverse effects on resistance and cooperation. If *α* is sufficiently high (0% agar) *P*_*CRIT*_ is reduced. Both cooperation and resistance are enhanced. While *δ* remains low under this condition, the increased access to nutrients results in more growth at *μ*_*o*_. This increased growth rate of the population, coupled with increased stability of AHL, reduces *P*_*CRIT*_. An intermediate value of *α* (0.2% agar) leads to a decrease in *P*_*CRIT*_. Cooperation is enhanced, but resistance is reduced. Here, increased access to AHL, coupled with an intermediate value of *δ* serves to reduce *P*_*CRIT*_. Note that resistance is reduced as *P*_*CRIT*_ changed from the undisturbed control. Finally, for low *α* (0.4% agar), *P*_*CRIT*_, and thus resistance and cooperation, remains relatively unchanged. Here, *δ* is already sufficiently high that increased access to AHL has minimal impact on cooperation.

Experimentally, we increased AHL access by growing our bacteria in medium with reduced pH. This serves to reduce the degradation rate of AHL but does not affect growth rate or GFP expression^42^. This indicates that changing pH in this study would predominantly affect AHL stability, and thus expression of genes under the regulation of AHL, such as the expression of CcdA. Our experimental results validate modeling predictions (Fig. 5E,F). Overall, these results suggest that while increased AHL access can enhance cooperation, it can simultaneously reduce resistance to periodic spatial disturbance.

**Figure 6:**
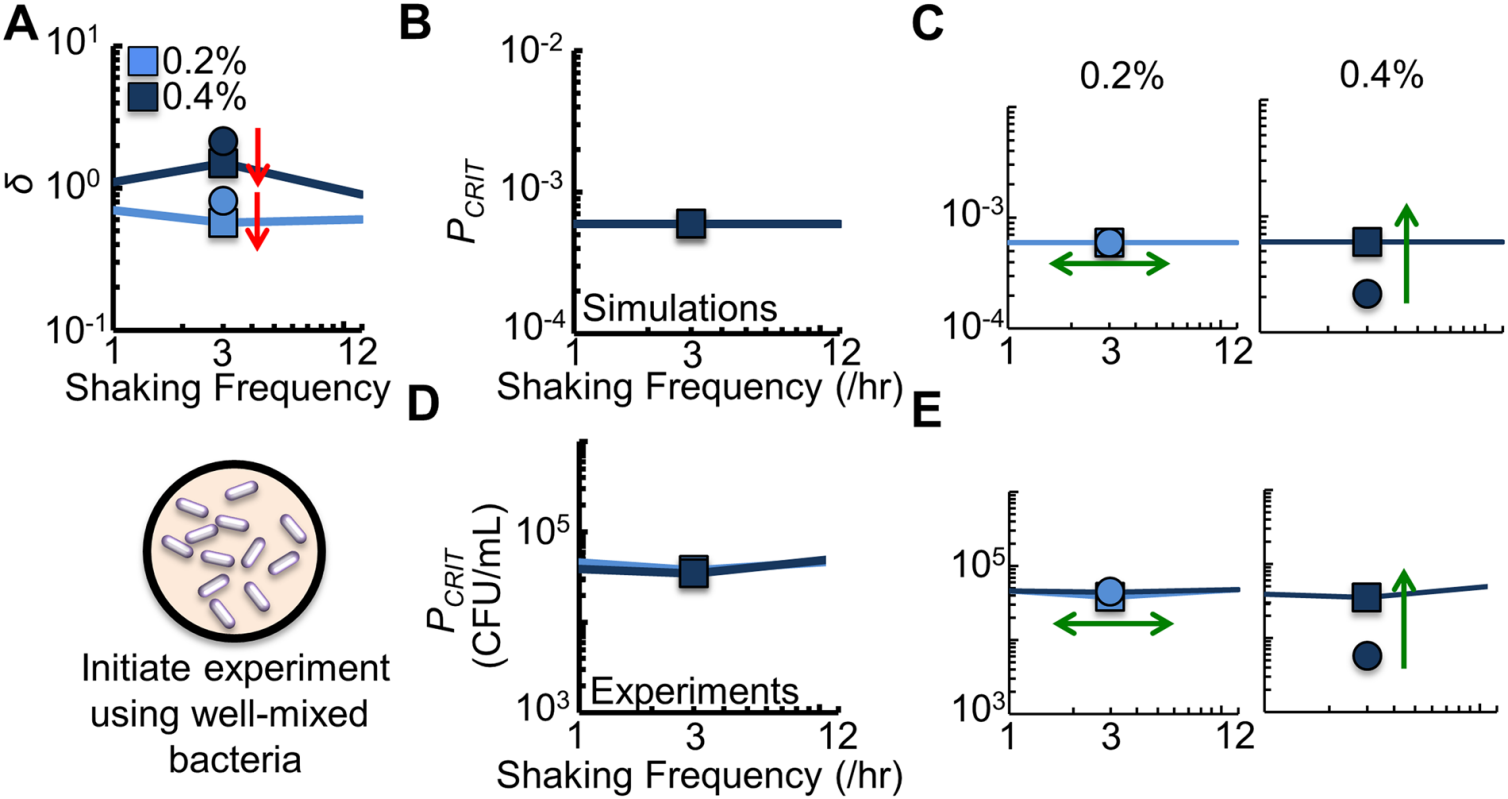
Decreasing access to AHL can increase resistance but reduces cooperation. (**A**) To reduce access to AHL in the model, we decreased *δ* by 0.7 at 3/hr (relative to *δ* presented in Fig. 2E). Experimentally, we mixed the bacteria in the well at the beginning of the experiment. For all panels, squares indicate decreased *δ*. Circles represent data re-plotted from Fig. 4. (**B**) Simulations results (Equations (1–3)) showing *P*_*CRIT*_ with decreased *δ*. For simplicity, we show the three shaking frequencies measured experimentally. (**C**) The result of individual simulations for each shaking frequency examined. For panels (C,E), green arrows indicate resistance increased (single ended arrow) or remained unchanged (double ended arrow). Direction of arrow indicates perturbation to *P*_*CRIT*_. (**D**) *P*_*CRIT*_ of engineered bacteria that were initially well-mixed in the microplate well. Two-tailed t-tests for each *P*_*CRIT*_ in Supplementary Tables S1 and S2, including comparisons between the well-mixed and non well-mixed initial conditions. SD from a minimum of three biological replicates. (**E**) Individual experiments for each agar density. Raw data in Supplementary Fig. S6.

To further investigate this tradeoff in our system, we reduced AHL access. To accomplish this mathematically, we decreased *δ* (Fig. 6A). Experimentally, we mimicked this reduction in AHL access (for 0.2% and 0.4% agar) by mixing the bacteria in the well at the beginning of the experiment. Our model predicts (Fig. 6B,C) and experiments validate (Fig. 6D,E) that *P*_*CRIT*_ increases if *α* is sufficiently low (0.4% agar). Although mixing reduced bacterial clustering, and thus AHL access, it simultaneously increased access to nutrients. This leads to more bacteria growing at a rate *μ*_*o*_, which enhances overall population growth. This manipulation served to increase resistance but reduce cooperation. However, if *α* was sufficiently high (0.2% agar), decreasing AHL access impacts *P*_*CRIT*_ minimally. Here, any increased access to nutrients was counterbalanced by a decrease in bacterial clustering, and thus access to AHL. As such, cooperation and resistance were unaffected. Taken together, our results emphasize a fundamental tradeoff: increased AHL access enhances cooperation but can simultaneously make the population less resistant to periodic spatial disturbances.

## Discussion

We have demonstrated that the frequency of periodic spatial disturbance can alter the ability of bacteria with a strong Allee effect to grow and survive. This was predominantly observed at an intermediate frequency of spatial disturbance (Fig. 4). In line with our results, previous studies have demonstrated that microbial diversity is highest^32^ and that cooperation is more stable in the presence of non-cooperating ‘cheater’ bacteria^20, 57^ at intermediate disturbance frequencies. These studies did not explicitly take into account spatial structure. Nevertheless, when taken together, it appears that intermediate disturbance frequency is an important feature affecting bacterial cooperation.

We realized periodic spatial disturbance by using the linear shaking function of the microplate reader. Previous studies have observed that agar densities of 0.5% or less may introduce viscoelastic properties to the medium^58^, which may introduce anisotropic flows in the medium along the direction of motion. In turn, this could bias the overall distribution pattern of bacteria, and disrupt any swimming motion of bacteria during shaking. However, shaking is the primary driver of movement in our system (Fig. 2B,C). Active swimming of bacteria is likely negligible as previous studies have shown that *E*. *coli* strain DH5αPRO (the strain which harbors the circuit) has a significantly reduced motility via swimming^59^. Finally, we did not observe growth patterns that were exclusively orientated with the direction of motion (Fig. 3B). Thus, anisotropy likely did not play a major role in determining our findings. It is possible that different patterns (e.g., orbital shaking) of shaking may alter the distribution of bacteria, and thus cooperation, in a different fashion than what is observed in this study. This remains an open and interesting question.

We found that by altering AHL access, the ability of a population to resist periodic spatial disturbance could be perturbed. This was most pronounced at intermediate disturbance frequency. Here, in 0.2% agar, increasing AHL access (pH 7.0) reduced resistance, while simultaneously enhancing cooperation. Resistance, and thus ecological stability, was reduced as *P*_*CRIT*_ was reduced from the undisturbed control condition (~10^4^ CFU/mL). Cooperation was enhanced as *P*_*CRIT*_ was reduced relative to bacteria grown in medium with 0.2% agar and at pH 7.4. While enhanced cooperation could benefit an individual population with a strong Allee effect, as it would reduce the density required to survive, it could also reduce the ecological stability of the environment where the population resides. Maintaining ecological stability often involves balancing species abundance and composition^28, 54^. By facilitating the growth of one species in an environment, species abundance may be perturbed. This disruption could undermine the long-term stability of the community, thus reducing the ability of the ecosystem to resist future disturbances. A direct experimental test of this notion would require the use of a microbial consortium.

Our results may have implications in the treatment of infectious diseases. Specifically, as our results demonstrate that the bacterial population requires a higher *P*_*CRIT*_ to initiate cooperation at an intermediate frequency of disturbance and fast dispersal (0% agar), it is plausible that cooperative pathogens may be less prone to initiating quorum sensing regulated pathogenesis if disturbed at an appropriate, intermediate frequency.

## Methods

### Strains and Growth Conditions

*Escherichia coli* strain DH5αPRO (Clontech, Mountain View, CA) was used in this study unless otherwise indicated. All experiments were performed in modified M9 medium [1X M9 salts (48 mM Na_2_HPO_4_, 22 mM KH_2_PO_4_, 862 mM NaCl, 19 mM NH_4_Cl), 0.4% glucose, 2% casamino acids (Teknova, Hollister, CA), 0.05% thiamine (Alfa Aesar, Ward Hill, MA), 2 mM MgSO_4_, 0.1 mM CaCl_2_] buffered to pH 7.0 or 7.4 with 100 mM 3-(N-morpholino)propanesulfonic acid (MOPS, Amresco, Solon, OH) with or without 0.2% or 0.4% agar (Alfa Aesar, Ward Hill, MA). 25 μg/mL chloramphenicol (Alfa Aesar, Heysham, England) and 50 μg/mL kanamycin (Amresco, Solon, OH) were used as appropriate. When growing bacteria in the microplate, the medium was overlaid with 70 μL of mineral oil (Fisher Scientific, Fair Lawn, NJ) to prevent evaporation. All experiments were initiated using single colonies that were inoculated into 5 mL of Luria-Bertani (LB) broth (MP Biomedicals, Solon, OH) containing the appropriate antibiotics. Liquid cultures were shaken at 37 °C for 24 hours at 250 RPM. Activation of the gene circuit was achieved by adding of 1 mM IPTG (Promega, Madison, WI). Transformations were performed using a Zymo Z-competent transformation kit (as per manufacturer’s specifications, Genesee Scientific, San Diego, CA).

For microscopy, we used *E*. *coli* strain DH5αPRO transformed with a plasmid containing *gfp*(*mut3b*)^60^ (colE1 origin of replication, chloramphenicol resistance, *gfp* driven by an anhydrotetracycline (atc) inducible *P*_*tet0*-*1*_ promoter^61^).

### Dispersal Rate of Bacteria

To determine the dispersal rate of the bacteria, we used a cell chamber (Ibidi, Martinsried, Germany) made of uncoated hydrophobic glass. Bacteria expressing *gfp* were grown overnight in 5 mL of LB with 25 μg/mL chloramphenicol at 37 °C. The following day, bacteria were resuspended in M9 medium containing 100 ng/mL of anhydrotetracycline (atc, Acros Organics, Geel, Belgium) and chloramphenicol and shaken for 90 minutes at 37 °C to induce GFP expression. 10 μL of the culture was inoculated at one end of the cell chamber, which contained 150 μL of M9 medium (containing 0%, 0.2% or 0.4% agar). Both openings of the cell chamber were then overlaid with 15 μL of mineral oil. After 20 minutes, the initial position of the leading edge of the bacterial population was observed and the position was recorded using an Olympus IX73P2F fluorescent microscope at 25X magnification with a DP-80 camera (Olympus Microscopes, Center Valley, PA) using the fluorescein isothiocyanate (FITC) and phase contrast filters. The chamber was then allowed to incubate at 37 °C without shaking on a level surface. For bacteria grown in 0.2% and 0.4% agar, the position of the leading edge was measured every 12 hours for 24 hours. For bacteria grown in 0% agar, the position of the leading edge was measured after 4 hours. cellSens software (Olympus Microscopes) was used to quantify the distance that the leading edge of the bacterial population travelled. Distances were averaged from three biological replicates. The dispersal rate was calculated by taking the total distance moved and dividing through by the number of hours over which the experiment occurred.

### Distance Travelled due to Shaking

*E*. *coli* expressing *gfp* were grown in 5 mL of LB medium containing 25 μg/mL chloramphenicol. The following day, the bacteria were resuspendend into M9 medium containing 25 μg/mL chloramphenicol and 100 ng/mL of atc and were shaken at 37 °C for 3 hours. 150 μL of M9 medium (containing 0%, 0.2% or 0.4% agar) was placed into a cell chamber. 10 μL of the bacteria was then inoculated into one of the ends of the chamber. The plate was left at room temperature for 10 minutes whereupon the initial position (leading edge) of the bacteria was recorded using an Olympus IX73P2F fluorescent microscope (25X magnification using a DP-80 camera). The plate was then placed in the microplate reader, secured to the robotic platform using tape, and shaken once as described in ‘*Spatial disturbance assays*’. The chamber was placed parallel to the shaking axis of the microplate reader. After removing the chamber from the microplate reader, the distance that the bacteria were displaced in the cell chamber was calculated using cellSens software. Distances were averaged from three biological replicates. For consistency both, the same cellSens software tool and approach were used to measure distance travelled in both ‘*Dispersal Rate of Bacteria*’ and ‘*Distance Travelled due to Shaking*’.

### Measuring nutrients in medium with 0% and 0.4% agar

To measure how agar density impacts growth and nutrient access, we inoculated ~10^6^ CFU/mL of engineered bacteria into the center of a 6-well plate containing 5 mL of M9 medium. This medium did not contain IPTG and contained either 0 or 0.4% agar. Next, we overlaid the medium with 2 mL of mineral oil and incubated the plate at 37 °C without shaking. After 24 hours, we removed four, 200 μL aliquots of medium from either the initial point of inoculation (0 cm) or 0.5 cm away from the initial point of inoculation. Note that, in 0% agar, bacterial growth appeared to be evenly distributed amongst the well, and was therefore equal at both sampling positions. In contrast, in 0.4% agar, bacterial growth was predominantly confined to the initial point of inoculation. Here, bacterial growth appeared to be reduced at the 0.5 cm sampling positions.

We pooled the aliquots from each sampling position and agar density. We filtered the pooled medium through a 0.22 μm syringe filter (PES, Genesee Scientific) to remove bacteria. We then inoculated ~10^6^ CFU/mL engineered bacteria from a fresh overnight culture into 50 μL of the filtered medium. This medium was then placed in a 96 well microplate and was overlaid with 70 μL of mineral oil. The microplate was placed in a VICTOR X4 microplate reader that was preheated to 37 °C. OD_600_ was measured every 20 minutes. After 16 hours, we plotted the final OD_600_ as a function of distance from the initial point of inoculation. Lower values of OD_600_ at 16 hours would indicate that less nutrients was available to the bacteria (see Supplementary Results for additional explanation).

### Spatial disturbance assays

*E*. *coli* containing the circuit^42^ were grown overnight in LB medium with chloramphenicol and kanamycin at 37 °C. The following day, a 10-fold dilution series was performed in M9 medium. CFUs were measured for all dilutions at the beginning of each experiment as previously described^42^. 190 μL of modified M9 medium (pH 7.4 or 7.0 using 100 mM MOPS) with different agar densities (0%, 0.2%, and 0.4%) with or without 1 mM IPTG was placed into a 96 well plate (REF 25-104; Olympus, San Diego, CA). 10 μL of each dilution was added to the center of the well. Each well was overlaid with 70 μL mineral oil. For bacteria grown with the circuit on, 1 mM of IPTG was added and vortexed before it was added the medium containing IPTG. The plate was then incubated in a PerkinElmer Victor X4 (Waltham, MA) microplate reader at 37 °C for 48 hours. The plate was shaken linearly at different frequencies for 10 seconds using a linear pattern with a radius of 0.1 mm. Shaking was performed on the fast setting, which has a frequency of 4800 mm/min and shakes the plate along the x-axis (or lengthwise). Measurements were taken at OD_600_ every 20 minutes for samples shaken at 3/hr, 6/hr, 9/hr and 12/hr, and every 60 minutes for samples shaken 1/hr. For experiments in the undisturbed environment, we incubated the plate in the microplate reader and measured OD_600_ every 20 minutes. However, we did not periodically shake the plate (absence of linear shaking) in the microplate reader. Here, to determine *P*_*CRIT*_, we averaged OD_600_ from bacteria grown in medium with 0%, 0.2% and 0.4% agar. For experiments initiated using the well-mixed condition, bacteria were resuspendend in M9 medium (containing either 0.2% or 0.4% agar) and were subsequently added to the wells of the microplate. A minimum of three biological replicates were performed. 48 hours was previously shown to be sufficient to determine if growth would occur for a given initial density and that ‘circuit blind’ mutants are unlikely to account for the majority of growth in the system^42^. To limit the formation of biofilms, we used a strain of bacteria (derivative of DH5α) that does not form robust biofilms^62^.

To determine growth rates, an overnight culture of bacteria was diluted 100-fold (~10^6^ CFU/mL) and was grown without IPTG as described above. We plotted OD_600_ as a function of time and determined the area of each curve that was representative of exponential growth. We fit exponential curves through a region that encompassed a change of at least 0.1 OD_600_ and had a R^2^ > 0.96 (majority > 0.98). We then averaged the slope of these fitted curves to determine the average growth rate. Growth rate was plotted from three biological replicates.

### Microscopy

To estimate the clustering of bacteria in the well, *E*. *coli* containing *gfp*(*mut3b*) were grown in the microplate reader as described in “*Spatial disturbance assays*”. However, the M9 medium contained only chloramphenicol and contained 100 ng/mL of anhydrotetracycline (atc) to induce expression of *gfp*. Bacteria were imaged at 0, 24 and 48 hours using an Olympus IX73P2F fluorescent microscope at 4X magnification.

The volume of clustered bacteria was estimated using cellSens software. While bacteria were distributed throughout the height of the well, we chose to quantify bacteria at the bottom of the well, as it was the most reliable plane to image, and could be consistently applied amongst all conditions and images.

For bacteria grown in medium with 0% agar, we first quantified the area (μm^2^) occupied by bacteria. To accomplish this, we used fifteen squares of the same size to designate regions of interest (ROI). We ensured that these ROIs were not placed over top of over saturated areas but were otherwise randomly placed on the image. We used cellSens software to calculate the total surface area (μm^2^) occupied by green fluorescence in these ROIs. Next, we used this surface area to estimate the total diameter of bacteria in the well by approximating the shape that the bacteria occupied as a circle. Here, we chose to approximate to a circle, as the shape of the well is cylindrical. Note that, we confirmed that bacteria were present from the center of the well to the edge of the well.

For bacteria grown in medium with 0.2% and 0.4% agar, we measured the circumference of the central cluster by drawing a line around the periphery using the freehand polyline measurement tool in cellSens software. We did not explicitly measure any small, organized colonies outside of this area. We used cellSens software to calculate the diameter (μm) of the central cluster by approximating its shape as a circle.

Next, we approximated the total volume (μm^3^) occupied by the central cluster using the equation for a cylinder. We approximated clustered cells to a cylinder in 0.2% and 0.4% agar, since the central cluster traversed the height of the well, and was approximated as a circle at the base. We applied the same approximation to cells in 0% agar for consistency. We note that the bacteria appeared to occupy space in the entire well, and the well shape is itself cylindrical.

We used the same approach to estimate the total volume at 0, 24 and 48 hours. Note that we could not reliably image the bacteria in every focal plane. We did not use automated segmentation, as the software could not reliably identify fluorescent signals in one plane, nor individual bacteria, in our experimental setup. Volumes are calculated based on a minimum of three biological replicates.

### Mathematical Modeling

We modeled our experimental system using three delayed differential equations. We model the global production of AHL, and the growth of bacteria both inside (*P*_*c*_) and outside (*P*_*o*_) of clusters.

1 $$\frac{dA}{dt}=\delta {k}_{p}({P}_{c}+{P}_{o})-{k}_{d}A$$

2 $$\frac{d{P}_{c}}{dt}=(\beta {P}_{o}-\alpha {P}_{c})+{\mu }_{c}{P}_{c}(1-{P}_{c}-{P}_{o})-\frac{\gamma {P}_{c}}{A(t-\tau )+{K}_{A}}$$

3 $$\frac{d{P}_{o}}{dt}=\,(\alpha {P}_{c}-\beta {P}_{o})+{\mu }_{o}{P}_{o}(1-{P}_{c}-{P}_{o})-\frac{\gamma {P}_{o}}{A(t-\tau )+{K}_{A}}$$

where *P*_*c*_ represents the population density in clusters (normalized to carrying capacity, unitless), *P*_*o*_ represents the population density outside of clusters (normalized to carrying capacity, unitless), *A* represents AHL (μM), *k*_*p*_ represents the clustering-dependent synthesis rate of AHL (μM/hr), *k*_*d*_ represents the degradation rate of AHL (/hr), *δ* represents the AHL production scalar due to bacterial clustering as a function of dispersal (unitless), *α* represents the dispersal rate of the bacteria from *P*_*c*_ to *P*_*o*_ (unitless), *β* represents the dispersal rate of bacteria from *P*_*o*_ to *P*_*c*_ (unitless), *μ*_*c*_ and *μ*_*o*_ represent the maximum growth rate of the bacteria inside and outside clusters (/hr), respectively, *γ* represents the killing rate of CcdB (μM/hr), *K*_*A*_ represents the half maximal killing ability of CcdB (μM), and *τ* represents the time delay of the activation of gene expression driven by the AHL-LuxR complex (hr). All simulations were completed in MATLAB (7.11.0 R2010b, The MathWorks Inc., Natick, MA) using the dde23 solver.

To determine *P*_*CRIT*_ from our model, we plotted the highest initial *P* that does not lead to an increase in total *P* over 5000 hours. Parameters for the model are presented in Supplementary Table S3. All simulations were performed for 5000 hours; under these conditions, the steady state has been reached.

### Estimation of *δ*

To estimate bacterial clustering, *δ*, we assumed that *δ* would be a function of the change in the volume of clustered bacteria normalized by the volume over which the bacterial population is spread. Previous studies have shown that AHL access depends on both local bacterial density (e.g., refs 9, 53, 63) and cell-cell proximity (i.e. spread, refs 64–66). As such, *δ* can be estimated as:

4 $$\delta =(\frac{{v}_{f}}{{s}_{f}})/(\frac{{v}_{i}}{{s}_{i}})$$

where *v*_*f*_ is the final volume occupied by clustered bacteria (μL), *v*_*i*_ is the initial volume occupied by clustered bacteria (μL), *s*_*f*_ is the final volume over which the bacteria (clustered and unclustered) were spread (μL), and *s*_i_ is the final volume over which the bacteria were spread (μL). Overall, *δ* can be thought of as the change in volume of the clustered bacteria normalized by the potential spreading volume due to agar density limitations. We chose to estimate *δ* based on these variables as both the volume of the clusters as well as the spreading boundaries (which would affect density) capture the spatial components of our system.

We note that although *δ* is a simplification of the distinct contributions of each population, it serves to accurately account for a weighted contribution of AHL synthesis. In particular, consider the extreme case where bacteria are highly clustered. In this case, *δ* will be sufficiently large, and *P*_*0*_ sufficiently small. Thus, contribution through AHL production will be distributed between the two populations proportional to their respective densities. In other words, non-clustered bacteria will contribute very little to the overall AHL accumulation. Instead, if *δ* is infinitely small, this implies that either ${v}_{i}\gg {v}_{f}$, or ${s}_{f}\gg {s}_{{\rm{i}}}$. The former is not biologically feasible given the setup of our system, and thus we focus on the latter case. If *s*_*f*_ is sufficiently large, then the bacteria are so far spread out that AHL production is minimal. This would serve to decrease the overall AHL accumulation of the system, and as a result, significantly increase *P*_*CRIT*_. We note that although AHL production on a per cell basis is unlikely to change with a large *s*_*f*_, this simplification accounts for the decrease of the overall AHL production of the population, and thus cooperation, due to clustering. Therefore, *δ* is a scalar simplification that accounts for the benefit of bacterial clustering on survival in our two-dimensional model.

To estimate the qualitative trends in volume occupied by clustered bacteria (*v*_*f*_ and *v*_*i*_), we used fluorescent microscopy to examine the spatial structure of *gfp*-expressing bacteria grown in our experimental setup (as described in “*Microscopy*”). Note that we performed these experiments with bacteria that do not contain the circuit to determine how shaking, and not the combination of shaking and AHL-mediated survival, affects the spatial distribution of cells. Coupling the measurement of these two variables would obscure the true contribution of shaking/dispersal alone in the system. A previous study has demonstrated that cooperation can drive spatial organization^18^. Our measurements may serve to underestimate the degree of clustering in bacteria when the circuit is activated, but this will not serve to drastically alter the overall trend. Experimentally, we estimated the *v*_*i*_ and *v*_*f*_ using cellSens software (see “*Microscopy*”). Qualitatively similar trends were observed at 24 and 48 hours.

Next, we estimated the change in the total volume over which the bacteria were spread. To simplify this estimation, we estimated and used a single ratio of $(\tfrac{{s}_{i}}{{s}_{f}})$ for each agar density. To estimate *s*_*i*_, we considered that at the beginning of the experiment (prior to bacterial dispersal in the medium), interactions between the bacteria would occur over the same volume. We assigned a value of 1.25 μL to *s*_*i*_, which is estimated based on the total volume initially occupied by the bacteria across all conditions (average *v*_*i*_ = 3.25 μL ± 2.00).

Over the course of the experiment, the volume over which bacteria both within and outside of clusters are spread increases. To estimate *s*_*f*_, we averaged *v*_*f*_ within each agar density. We then multiplied this value by 1.25 to account for the volume occupied by bacteria outside of clusters. This value of 1.25 was estimated based on our microscope images with 0% agar. Here, the average *v*_*f*_ across each shaking frequency was 172.6 μL ± 14.1. However, bacteria were spread out across the entire ~200 μL well volume, representing a ~25% increase over the volume of clustered bacteria. Therefore, we estimated *s*_*f*_ for this condition as 198.5 μL. Using this approach, we estimated *s*_*f*_ for 0.2% agar and 0.4% agar as 13.5 (average *v*_*f*_ = 11.7 ± 8.9) and 6.1 (average *v*_*f*_ = 5.3 ± 4.8), respectively. Overall, we find the value of $(\tfrac{{s}_{i}}{{s}_{f}})$ to be ~0.006, ~0.09 and ~0.2 (±one order of magnitude to account for error in measurements) for media with agar densities of 0%, 0.2% and 0.4%, respectively. We could not reliably measure the entire volume of both clustered and unclustered bacteria as measuring unclustered bacteria in every focal plane was not possible in our experimental setup. As such, we had to estimate *s*_*f*_ as described above.

Using the above estimations of *v*_*f*_, *v*_*i*_, *s*_*i*_, and *s*_*f*_, we plotted *δ* as a function of shaking frequency (Fig. 4E, left panel). Next, we fit *δ* in our model to this general trend (Fig. 4E, right panel) according to a second order polynomial function *δ* = *p*_1_ *x* ^2^ + *p*_2_ *x* + *p*_3_, where *x* is the logarithmically transformed shaking frequency (consistent with the x-axis in Fig. 2C). We note that decreasing *δ* with increasing shaking does not predict our experimental results (Supplementary Fig. S3).

The estimation of all other parameters can be found in the Supplementary Results section.

### Statistical Analysis

Unless otherwise indicated, we used a student’s two-tailed t-test to calculate *p* values (*p* ≤ 0.05). To determine *P*_*CRIT*_, a two-tailed t-test was used to determine if the OD_600_ values observed at 48 hours were statistically different from zero. In cases where the OD_600_ value was less than 0.01, the value was set to zero as this is below the detectability level of our instrument and likely represents minor differences in background readings. This assumption has been used in previous studies to carry out similar analyses^42^. *P*_*CRIT*_ was reported as the highest initial density where the OD_600_ value at 48 hours was not statistically different than zero. To determine if growth rates were different in the various shaking frequencies, a two-tailed t-test was performed between the slopes as calculated above.

## Electronic supplementary material

Supplementary information
