# Supplementary material for: Cooperation and competition shape ecological resistance during periodic spatial disturbance of engineered bacteria
Source: Sci Rep. 2017 Mar 27;7:440. doi: 10.1038/s41598-017-00588-9 (PMC5428654; doi:10.1038/s41598-017-00588-9)

SUPPLEMENTARY INFORMATION

Cooperation and competition shape ecological resistance during periodic spatial disturbance of engineered bacteria

Cortney E. Wilson[1,2], Allison J. Lopatkin[3], Travis J.A. Craddock[4-7], William W. Driscoll[8], Omar T. Eldakar[1], Jose V. Lopez[1,2] and Robert P. Smith[1,*].

[1] Department of Biological Sciences, Halmos College of Natural Sciences and Oceanography, Nova Southeastern University, Fort Lauderdale, FL, 33314

[2] Guy Harvey Oceanographic Center, Nova Southeastern University, Dania Beach FL, 33004

[3] Department of Biomedical Engineering, Duke University, Durham, North Carolina, USA

[4] Clinical Systems Biology Group, Institute for Neuroimmune Medicine, [5] Department of Neuroscience, College of Psychology, [6] Department of Computer Science, College of Engineering and Computing, [7] Department of Clinical Immunology, College of Osteopathic Medicine, Nova Southeastern University, Fort Lauderdale FL, 33314

[8] University of Minnesota, Department of Ecology, Evolution, and Behavior, St. Paul, MN 55108

*Correspondence should be addressed Robert P. Smith. E-mail: rsmith@nova.edu Tel: 954 262 7979

SUPPLEMENTARY RESULTS

Growth rate is altered by agar density, not shaking frequency

Towards offering an explanation for the reduction in growth rate as agar density increases (Fig. 4B), bacteria were grown in medium with 0% or 0.4% agar (Fig. 4C). After 24 hours, media taken 0.5 cm away and overtop of the initial point of inoculation (0 cm) was filtered. Bacteria from a fresh overnight culture were inoculated in the filtrate and OD_{600} was examined after 16 hours of growth at 37°C. In medium with 0% agar, the bacteria grew to equal OD_{600} at both positions (0 cm and 0.5 cm, $p = 0.574$). In medium with 0.4% agar, the bacteria grown in medium taken 0.5 cm away from the initial point of inoculation grew significantly more than those grown in medium taken from the initial point of inoculation (0 cm, $p = 0.004$). It can be hypothesized that this reduction in bacterial growth is indicative of less nutrients available at this position due to increased competition. Additional studies have found that nutrients are reduced and can become limiting in the center of a bacteria cluster/colony [1-3]. As we overlaid each well with mineral oil to prevent evaporation in our spatial disturbance assays, it is unlikely that oxygen consumption and accessibility is a driver in the trends that we observed in our experiments [4].

We note that there is a difference in OD_{600} at 0 cm between media with 0% agar (0.135 ± 0.0018) and 0.4% agar (0.183 ± 0.013, $p = 0.021$). This may reflect an increased carrying capacity or growth rate due to the production of secondary metabolites or additional modifications to the medium. These secondary metabolites or modifications to the medium would be more concentrated in the 0.4% agar condition at 0 cm, as there are more bacteria located at this position relative to the 0% agar condition. It may also represent an increased OD_{600} value due to clustering of bacteria as the agar density of the medium is higher than 0% agar. Overall, however, this would not influence the conclusions of the manuscript, nor the overall trends in our simulations.

As our microplate takes measurements directly in the center of the well, measurements in medium with 0.2% and 0.4% agar are examining growth rate of the central cluster. As bacteria are more confined to the center of the well under this condition, OD_{600} may artificially increase faster (as opposed to a well mixed population observed in 0% agar where bacteria growth occurs throughout the environment). This could serve to overestimate the growth rate of bacteria grown under this condition, but would not affect the general trend in the data (bacteria in 0.2% and 0.4% agar grow more slowly than when in 0% agar) nor our modeling assumptions.

Model development and assumptions

We assume that AHL diffusion is faster than growth or gene expression, and that transport of AHL across the cell wall and membrane is sufficiently fast such that the concentration of AHL on the outside of the bacteria is equal to that on the inside [5]. Furthermore, we assume that agar density does not change the rate of AHL diffusion. This assumption is justified as previous studies have observed that increasing agar density (within the range in this study, 0%-0.4% agar) in medium does not have an observable effect on the diffusion rate of small molecules, including the AHL used in this study (e.g., [3,6]). The clustering-dependent synthesis rate of AHL (k_p) follows first order kinetics for AHL production, consistent with previous models (e.g., [7]). As intracellular AHL activates CcdA expression via LuxR, we assumed that expression of CcdA is linearly activated by the concentration of AHL. As the LuxR-AHL complex reaches quasi steady state quickly, we do not model this molecular species [8]. We explicitly model the decay of AHL using the term k_d [9].

Our model includes a time delay term, τ. This represents the time delay of the activation of gene expression by the LuxR-AHL complex [7]. Overall, τ is a lumped parameter that takes into account several reactions in the bacteria including the time required to synthesize CcdA, the time required for CcdA to bind CcdB and the time required for the bacteria to recover from CcdB

poisoning and begin growing [10]. It is not critical to incorporate the individual reactions to generate our qualitative results (See Supplementary Results - "*Nonlinear activation of CcdA*", Supplementary Fig. S3)

Our model assumes independent growth of two populations, P_c and P_o, which exist within and outside of clusters, respectively. Each population grows at a different maximal growth rate that is dependent upon the agar density (Fig. 4B). At higher agar density, we assume that the majority of bacteria stay in the clusters, which reduces the maximal growth rate of P_c due to nutrient limitation. Previous studies have found that highly clustered bacteria suffer from nutrient limitation, which serves to reduce growth rate (e.g. [2,3]), consistent with our data (Fig. 4C). Furthermore, a previous study has indicated that microbes initiated from densities as low as 10^3 CFU/mL suffer nutrient limitation in medium with high agar density due to increased clustering and reduced mobility [3]. Consequently, we assume that bacteria located outside of clusters will have access to more nutrients, and thus have a higher maximal growth rate. As such, bacteria in P_o grow faster relative to P_c but contribute less AHL due to the effects of reduced cooperation.

To incorporate the effect of bacterial clustering, we assume that the clustering-dependent synthesis rate of AHL (k_p) is proportional to bacterial clustering (δ) at the steady state in the environment according to first order kinetics. Previous studies have demonstrated that the spatial structure of bacteria effects density-dependent dynamics, such as cooperation. Specifically, bacteria within a cluster/colony experience a higher concentration AHL [1,11] and shared metabolites [3]. As our model does not account for spatial factors explicitly, in our system, this would serve to create a form of AHL driven positive feedback [12], where the presence of AHL drives the production of more AHL. The survival and division of these bacteria will go on to produce more AHL in the system, thus forming the positive feedback. Conversely, bacteria that have left the cluster through shaking of the microplate reader would experience, and thus produce, less AHL. Thus, δ serves as a scaling

factor that accounts for the average AHL synthesis rate across the spatial distribution of all cells in the population.

Overall, a large δ value reflects a highly clustered environment and results in more AHL synthesis. As the value of δ is reduced, bacterial clustering in the system is also reduced towards a spatially homogeneous distribution of bacteria, resulting in less AHL synthesis. Finally, we note that although diffusion for AHL is fast, previous studies have shown that despite fast diffusion, shared metabolites can still favor growth in clusters that are separated by less than ~10 mm [3]. That is, the volume of a well in a 96 well plate is sufficiently large to allow accumulation of metabolites in one defined area that is sufficient to benefit microbial growth.

We modeled the movement of bacteria due to shaking using two parameters, α and β. We note that the magnitude of these parameters is determined by both shaking frequency and agar density. We estimated that bacteria move, on average, 10-fold higher during each shake, and dispersal decreases with higher density agar confinement [3] (Fig. 2C). We do not explicitly account for diffusion of the bacteria, as this rate was significantly lower than movement due to shaking (Fig. 2B).

Finally, we assume that if P_c is below a numerical threshold (10^{-7}), then neither population can survive. Experimentally, this would represent approximately 10 CFU/mL (or 2 CFU in a 200 μL well), which is well below all P_{CRIT} observed experimentally.

Parameter Estimation

Parameters used in our mathematical model can be found in Supplementary Table S3. Where available, we used previously published data to estimate the magnitude of each parameter. Specifically, we used previously published data to estimate the values of killing rate of CcdB (γ) [7], the clustering-dependent synthesis rate of AHL (k_p) [13], K_A represents the half maximal killing ability of

CcdB (K_A) [7], the time delay of the activation of gene expression driven by the AHL-LuxR complex (τ) [7] and the degradation rate of AHL (k_d) [14]. The maximal growth rates μ_c and μ_o for each shaking condition were estimated using growth rates (Fig. 4B) and growth curves [7].

We estimated the value of α using the data presented in Fig. 2C. As agar density increased, the distance travelled by the bacteria decreased approximately ten-fold. We then assumed that as shaking frequency increased, the total distance travelled by the bacteria in an hour would increase. To model this dispersal trend, we fit the experimental data to a log-transformed linear regression, such that $log(\alpha) = r_1 x + r_2$, and x is the logarithmically transformed shaking frequency (consistent with the x-axis in Fig. 2C). Note that the change in α normalized by agar density demonstrates that dispersal is linear and relatively equal as shaking frequency increases (Supplementary Fig. S3). For simplicity, we assumed that β was 5% of α. Reasonable changes in β do not drastically affect the predictions of our model (Supplementary Fig. S4).

To manipulate AHL access, we chose to manipulate k_d in Equation (1) as previous studies have shown that increasing pH reduces the AHL degradation rate [15]. We chose not to directly perturb k_p as changes in pH should not directly influence transcription and translation of LuxI [7].

Sensitivity analysis for parameters β, r_1, r_2, p_1, p_2, p_3, K_a, γ, k_d, k_p and τ, is presented in Supplementary Fig. 4. We note that the majority of our modeling predictions are qualitatively robust to changes in these parameters when varied .5X and 2X the values presented in Supplementary Table S3.

Nonlinear activation of CcdA

Our model assumes that expression of CcdA is linearly dependent upon the concentration of AHL. This simplifying assumption can be made as previous studies have found that activation of gene expression using LuxR-AHL complexes is only mildly cooperative. Specifically, dose- (AHL)

response (*gfp* production) curves have demonstrated that AHL-induced GFP expression is only slightly cooperative, and relatively linear once AHL has reached a sufficient concentration (~ 1nM) to trigger gene expression [16,17]. Pai et al., used cooperatively values (in a Hill equation) that ranged from 1-2 to model AHL-induced gene expression [18]. Previous studies that have explicitly quantified the cooperativity value of LuxR-AHL have assigned values ranging from 0.85-1.6 [19,20]. Given the low magnitude of cooperativity, we simplified the relationship between AHL and CcdA to be linear.

Nevertheless, if CcdA expression is modeled as a nonlinear function of AHL, our results are qualitatively consistent with Fig. 4A. To demonstrate this, we altered Equations (2) and (3) as follows:

$$\frac{dP_c}{dt} = (\beta P_o - \alpha P_c) + \mu_c P_c(1 - P_c - P_o) - \frac{\gamma P_C}{K_A + \frac{A(t-\tau)^n}{A(t-\tau)^n + K_{AHL}^n}} \quad \text{Equation (S1)}$$

$$\frac{dP_o}{dt} = (\alpha P_c - \beta P_o) + \mu_o P_o(1 - P_c - P_o) - \frac{\gamma P_o}{K_A + \frac{A(t-\tau)^n}{A(t-\tau)^n + K_{AHL}^n}} \quad \text{Equation (S2)}$$

We used a cooperativity (*n*) value of 2, which is towards the higher end of cooperativity values reported in the literature [18]. K_{AHL} represents the represents the half maximal constant of AHL driven gene expression (μM). We set this value to 0.05. All other parameters as shown in Supplementary Table S3.

SUPPLEMENTARY REFERENCES

1 Hense, B. A., Kuttler, C. & Muller, J. *The Physical Basis of Bacterial Quorum Communication Biological and Medical Physics, Biomedical Engineering* (*Springer, 2015*).

2 Hense, B. A., Müller, J., Kuttler, C. & Hartmann, A. Spatial heterogeneity of autoinducer regulation systems. *Sensors* **12**, 4156-4171 (2012).

3 Ratzke, C. & Gore, J. Self-organized patchiness facilitates survival in a cooperatively growing *Bacillus subtilis* population. *Nature Microbiology*, 16022 (2016).

4 Wessel, A. K. *et al.* Oxygen Limitation within a bacterial aggregate. *mBio* **5**, e00992-00914, doi:10.1128/mBio.00992-14 (2014).

5 Kaplan, H. B. & Greenberg, E. P. Diffusion of autoinducer is involved in regulation of the *Vibrio fischeri* luminescence system. *Journal of Bacteriology* **163**, 1210-1214 (1985).

6 Song, H., Payne, S., Gray, M. & You, L. Spatiotemporal modulation of biodiversity in a synthetic chemical-mediated ecosystem. *Nature Chemical Biology* **5**, 929-935, doi:http://www.nature.com/nchembio/journal/v5/n12/suppinfo/nchembio.244_S1.html (2009).

7 Smith, R. *et al.* Programmed Allee effect results in a tradeoff between population spread and survival. *Proceedings of the National Academy of Science USA* **111**, 1969-1974 (2014).

8 Haseltine, E. L. & Arnold, F. H. Implications of rewiring bacterial quorum sensing. *Applied and Environmental Microbiology* **74**, 437-445, doi:10.1128/aem.01688-07 (2008).

9 Kauffman, M. J. & Jules, E. S. Heterogeneity shapes invasion: host size and environment influence susceptibility to a nonnative pathogen. *Ecological Applications* **16**, 166-175, doi:10.1890/05-0211 (2006).

10 Dwyer, D. J., Kohanski, M. A., Hayete, B. & Collins, J. J. Gyrase inhibitors induce an oxidative damage cellular death pathway in *Escherichia coli*. *Molecular Systems Biology* **3**, doi:http://www.nature.com/msb/journal/v3/n1/suppinfo/msb4100135_S1.html (2007).

11 Hense, B. A. *et al.* Does efficiency sensing unify diffusion and quorum sensing? *Nature Reviews Microbiology* **5**, 230-239 (2007).

12 West, S. A., Winzer, K., Gardner, A. & Diggle, S. P. Quorum sensing and the confusion about diffusion. *Trends in Microbiology* **20**, 586-594, doi:http://dx.doi.org/10.1016/j.tim.2012.09.004 (2012).

13 Ravn, L., Christensen, A. B., Molin, S., Givskov, M. & Gram, L. Methods for detecting acylated homoserine lactones produced by Gram-negative bacteria and their application in studies of AHL-production kinetics. *Journal of Microbiological Methods* **44**, 239-251, doi:http://dx.doi.org/10.1016/S0167-7012(01)00217-2 (2001).

14 Kaufmann, G. F. *et al.* Revisiting quorum sensing: discovery of additional chemical and biological functions for 3-oxo-N-acylhomoserine lactones. *Proceedings of the National Academy of Sciences of the United States of America USA* **102**, 309-314, doi:10.1073/pnas.0408639102 (2005).

15 You, L., Cox, R. S., Weiss, R. & Arnold, F. H. Programmed population control by cell-cell communication and regulated killing. *Nature* **428**, 868-871 (2004).

16 Collins, C. H., Arnold, F. H. & Leadbetter, J. R. Directed evolution of *Vibrio fischeri* LuxR for increased sensitivity to a broad spectrum of acyl-homoserine lactones. *Molecular Microbiology* **55**, 712-723, doi:10.1111/j.1365-2958.2004.04437.x (2005).

17 Collins, C. H., Leadbetter, J. R. & Arnold, F. H. Dual selection enhances the signaling specificity of the quorum-sensing transcriptional activator LuxR. *Nature Biotechnology* **24**, 708-712 (2006).

18 Pai, A., Tanouchi, Y. & You, L. Optimality and robustness in quorum sensing (QS)-mediated regulation of a costly public good enzyme. *Proceedings of the National Academy of Sciences* **109**, 19810-19815, doi:10.1073/pnas.1211072109 (2012).

19 Canton, B., Labno, A. & Endy, D. Refinement and standardization of synthetic biological parts and devices. *Nature Biotechnology* **26**, 787-793, doi:10.1038/nbt1413 (2008).

20 Urbanowski, M. L., Lostroh, C. P. & Greenberg, E. P. Reversible acyl-homoserine lactone binding to purified *Vibrio fischeri* LuxR protein. *Journal of Bacteriology* **186**, 631-637, doi:10.1128/jb.186.3.631-637.2004 (2004).

21 Pai, A. & You, L. Optimal tuning of bacterial sensing potential. *Molecular Systems Biology* **5**, 286, doi:msb200943 [pii] 10.1038/msb.2009.43 (2009).

SUPPLEMENTARY FIGURE LEGENDS

Supplementary Figure S1. Raw OD_{600} values of engineered bacteria grown for 48 hours at various shaking frequencies.

A.-C. When grown without IPTG, bacteria grew regardless of initial density and shaking frequency. Panel A = 0% agar, panel B = 0.2% agar, panel C = 0.4% agar. In all panels, data plotted from a minimum of three experiments.

D.-F. When grown with IPTG, bacteria did not grow if initiated below a specific initial density. Panel D = 0% agar, panel E = 0.2% agar, panel F = 0.4% agar. P_{CRIT} reported in Supplementary Table S1 along with statistical analysis. For statistical comparisons among values of P_{CRIT} under each condition, consult Supplementary Table S2.

Supplementary Figure S2. P_{CRIT} plotted as a function of δ.

A. P_{CRIT} from 1/hr, 3/hr, and 12/hr (from Fig. 4A) plotted as a function of experimentally measured δ (from Fig. 4E, left panel).

B. Simulation results of P_{CRIT} at 1hr, 3/hr, and 12/hr plotted as a function of δ fit from experiments (from Fig. 4E, right panel). Ten discrete points are plotted per agar density (values of α).

Supplementary Figure S3. Our mathematical model predicts the qualitative trends of our experimental data.

A. Simulations demonstrating how initial P ($P_c + P_o$) influences final P. Without activation of the circuit ($\gamma = 0$, off, blue line), final P increases regardless of initial P. With the circuit activated (on, red line), final P increases only if initial P is sufficiently high.

B. Simulations results with nonlinear activation of *ccdA* by AHL (Equations (S1) and (S2)). We assume $n = 2$, where n represents the cooperativity value.

C. Simulations showing α normalized by agar density. As shaking frequency increases, the dispersal rate increases relatively equally across conditions.

D. Simulations showing the effect of modeling δ as a decreasing function of increasing shaking frequency (left panel) and its effect on P_{CRIT} (right panel). The value of δ at 3/hr is decreased so that the relationship between δ and shaking frequency is linear.

Supplementary Figure S4. Sensitivity analysis of model parameters. Each parameter is varied 5 times between 0.5X (light blue) and 2X (dark blue) of the base value (Supplementary Table S3).

Supplementary Figure S5. Raw data demonstrating the effect of decreasing pH/k_d on P_{CRIT}.

A-C. Raw OD_{600} values of engineered bacteria grown in medium with IPTG (pH 7.0) for 48 hours. Panel A = 0% agar, panel B = 0.2% agar, panel C = 0.4% agar. In all panels, data plotted from a minimum of three experiments. P_{CRIT} reported in Supplementary Table S1 along with statistical analysis. For statistical comparisons among values of P_{CRIT} under each condition, consult Supplementary Table S2.

D. Simulations (500 initial cell densities and 500 shaking frequencies, Equations (1-3)) of P_{CRIT} with reduced k_d. The overall trends are qualitatively consistent with Fig. 5C-F. However, we were unable to observe a quantitative match in our experiments due to the inability to precisely control initial cell density.

E. Simulated differences in P_{CRIT} between 7.4 and 7.0 at 1/hr, 3/hr and 12/hr. Discrete data points plotted from simulation in panel D.

Supplementary Figure S6. Raw OD_{600} values of engineered bacteria grown for 48 hours at various shaking frequencies initiated under well-mixed conditions. Panel A = 0% agar, panel B = 0.4% agar. P_{CRIT} reported in Supplementary Table S1 along with statistical analysis. For statistical comparisons among values of P_{CRIT} under each condition, consult Supplementary Table S2. Data plotted from a minimum of three experiments.

SUPPLEMENTARY TABLES

Supplementary Table S1. *p* values for P_{CRIT} reported in this manuscript. Statistical significance calculated using a two-tailed t-test.

Density of Agar	Shaking Frequency (/hr)	pH	P_{CRIT} (CFU/mL)	Standard Deviation (CFU/mL)	*p* value
0%	1	7.4	3.68 x 10^4	1.86 x 10^4	0.07
	3		3.93 x 10^5	5.75 x 10^4	0.06
	6		3.98 x 10^5	1.24 x 10^5	0.08
	9		3.30 x 10^4	3.13 x 10^3	0.11
	12		3.85 x 10^4	3.07 x 10^3	0.14
0.2%	1		4.65 x 10^4	7.69 x 10^3	0.36
	3		4.47 x 10^4	1.30 x 10^4	0.33
	6		4.00 x 10^4	1.33 x 10^4	1.00
	9		3.30 x 10^4	3.13 x 10^3	0.08
	12		4.77 x 10^4	1.30 x 10^4	0.08
0.4%	1		3.88 x 10^4	1.01 x 10^4	0.25
	3		5.70 x 10^3	1.31 x 10^3	0.24
	6		1.70 x 10^4	8.08 x 10^3	0.39
	9		3.30 x 10^4	3.13 x 10^3	0.36
	12		5.10 x 10^4	1.50 x 10^4	1.00
0%	1	7.0	4.55 x 10^4	1.88 x 10^4	0.26
	3		4.73 x 10^4	1.44 x 10^4	0.20
	12		4.70 x 10^4	5.81 x 10^3	0.32
0.2%	1		4.09 x 10^4	1.46 x 10^4	0.08
	3		4.80 x 10^3	1.15 x 10^2	0.40
	12		4.68 x 10^4	5.60 x 10^3	0.07
0.4%	1		5.35 x 10^3	1.28 x 10^3	0.09
	3		4.80 x 10^3	1.15 x 10^2	0.39
	12		4.22 x 10^4	6.74 x 10^3	0.08
0.2%	well-mixed	7.4	3.78 x 10^4	2.76 x 10^3	0.08
0.4%			3.5 x 10^4	7.64 x 10^3	0.09
Calculated using 0%, 0.2%, and 0.4%	undisturbed	7.4	4.84 x 10^4	1.91 x 10^4	0.12

Supplementary Table S2. *p* values for comparison between P_{CRIT} achieved using various agar densities, shaking frequencies and pH.

Density of Agar (1)	Density of Agar (2)	Shaking Frequency (/hr)	pH		*p* value
0%	0.2%	1	7.4		0.13
		3			<0.001
		6			<0.001
		9			1.00
		12			0.15
0.2%	0.4%	1			0.17
		3			0.001
		6			0.01
		9			1.00
		12			0.73
0%	0.4%	1			0.76
		3			<0.001
		6			<0.001
		9			1.00
		12			0.19
0%	0.2%	1	7.0		0.57
		3			<0.001
		12			0.92
0.2%	0.4%	1			<0.001
		3			1.0
		12			0.06
0%	0.4%	1			0.001
		3			0.001
		12			0.10
0%	0%	1	7.4	7.0	0.32
		3			<0.001
		12			0.004*
0.2%	0.4%	1			0.31
		3			0.001
		12			0.87
0.4%	0.4%	1			<0.001
		3			0.09
		12			0.33
0.2%	0.2%	3	Center	Mix	0.25
0.4%	0.4%	3			<0.01
0%	0%	1	7.4 compared to undisturbed		0.15
		3			<0.001
		12			0.12
0.2%	0.2%	1			0.78
		3			0.64
		12			0.93
0.4%	0.4%	1			0.20
		3			<0.001
		12			0.2
0%	0%	1	7.0 compared to undisturbed		0.75
		3			0.88
		12			0.82
0.2%	0.2%	1			0.31
		3			<0.001
		12			0.78
0.4%	0.4%	1			<0.001
		3			<0.001
		12			0.33
0.2%	0.2%	3	7.4 compared to undisturbed		0.10
0.4%	0.4%	3			0.06

*While these two P_{CRIT} are statistically different, they are within the same order of magnitude ($3.85 \times 10^4 \pm 3.07 \times 10^3$ and $4.70 \times 10^4 \pm 5.81 \times 10^3$). We consider these values to indicate that P_{CRIT} did not change given that changes in other values of P_{CRIT} are approximately an order of magnitude apart, we are sampling from a 10-fold dilution series and this result is consistent with our modeling predictions.

Supplementary Table S3. Parameters used for mathematical modeling.

<table>
<tr><th>Parameter</th><th>Description</th><th colspan="4">Rate constant</th><th>Reference</th></tr>
<tr><td>μ_c</td><td>Maximum growth rate of bacteria in a cluster</td><td colspan="4">0% = 0.48/hr
0.2% = 0.44/hr
0.4%=0.44/hr</td><td>Estimated from Fig. 2B-C and [7].</td></tr>
<tr><td>μ_o</td><td>Maximum growth rate of bacteria outside of a cluster</td><td colspan="4">0.5/hr</td><td>Estimated from Fig. 2B-C and [7].</td></tr>
<tr><td>k_p</td><td>Clustering-dependent synthesis rate of AHL</td><td colspan="4">0.4 μM/hr</td><td>[13,21]</td></tr>
<tr><td>k_d</td><td>Degradation rate of AHL</td><td colspan="4">0.01/hr (pH 7.4 experiments)
0.001/hr (pH 7.0 experiments)</td><td>[14]</td></tr>
<tr><td rowspan="4">α</td><td rowspan="4">Dispersal rate of bacteria from P_0 to P_1 (ranges indicate lowest and highest value depending on shaking frequency)</td><td></td><td>r_1</td><td>r_2</td><td></td><td rowspan="4">Experimental data fit to equation $log(\alpha) = r_1 x + r_2$</td></tr>
<tr><td>0%</td><td>1.4106</td><td>-5.2621</td><td></td></tr>
<tr><td>0.2 %</td><td>1.3060</td><td>-7.9755</td><td></td></tr>
<tr><td>0.4 %</td><td>1.2680</td><td>-10.6678</td><td></td></tr>
<tr><td rowspan="4">δ</td><td rowspan="4">Bacterial clustering</td><td></td><td>p_1</td><td>p_2</td><td>p_3</td><td rowspan="4">Experimental data fit to equation $\delta = p_1 x^2 + p_2 x + p_3$</td></tr>
<tr><td>0%</td><td>0.0787</td><td>-0.2139</td><td>0.2445</td></tr>
<tr><td>0.2 %</td><td>- 0.1012</td><td>0.2116</td><td>0.7030</td></tr>
<tr><td>0.4 %</td><td>- 0.7445</td><td>1.7700</td><td>1.1034</td></tr>
<tr><td>γ</td><td>Killing rate of CcdB</td><td colspan="4">$4x10^{-3}$ μM/hr</td><td>[7]</td></tr>
<tr><td>K_A</td><td>Half maximal killing ability of CcdB</td><td colspan="4">$7x10^{-3}$ μM</td><td>[7]</td></tr>
<tr><td>β</td><td>Dispersal rate of bacteria from P_1 to P_0</td><td colspan="4">0.05 α</td><td>Estimated</td></tr>
<tr><td>τ</td><td>Time delay of the activation of gene expression by the LuxR-AHL complex</td><td colspan="4">7 hr</td><td>[7]</td></tr>
</table>

Supplementary Fig. S1

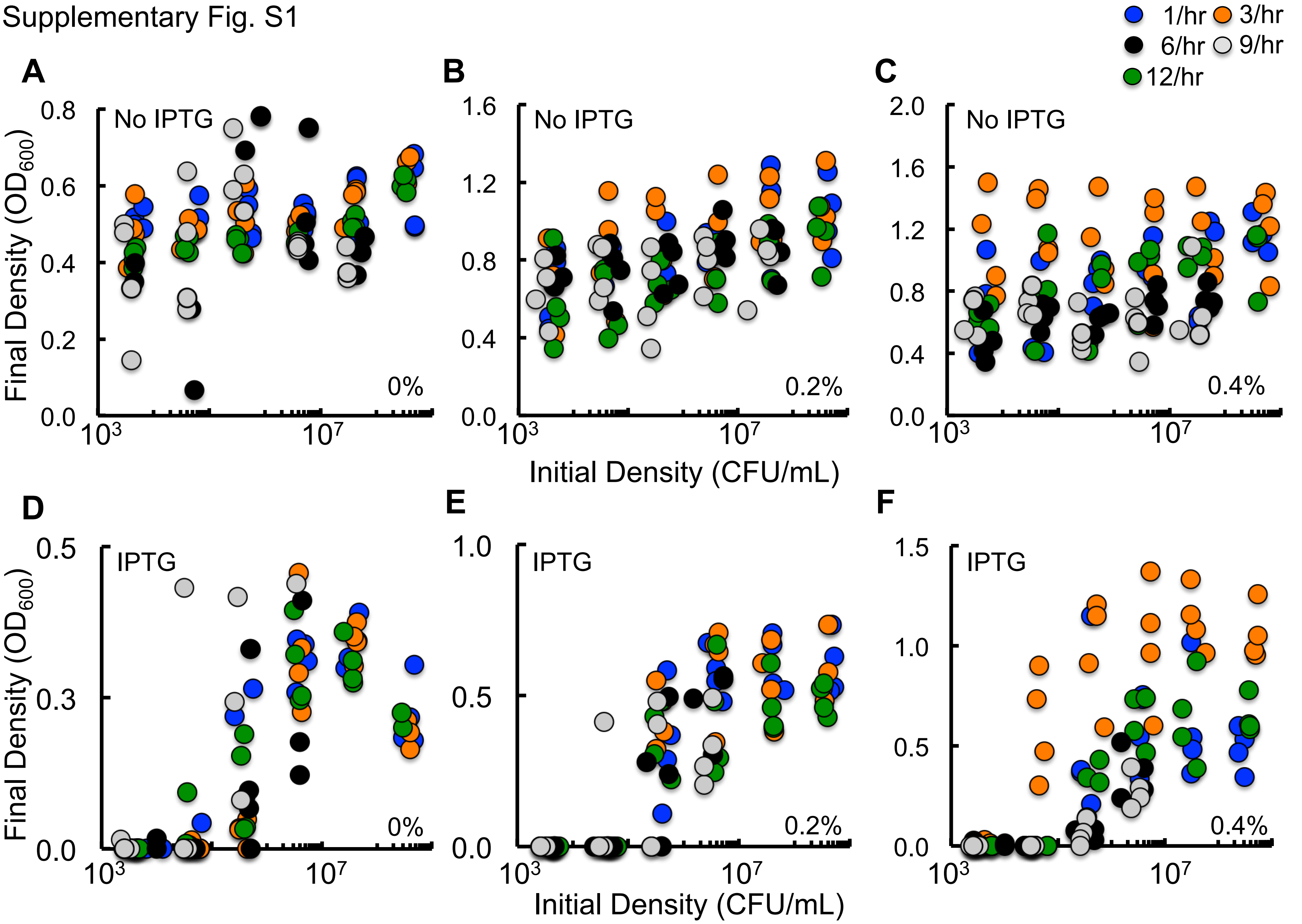

Supplementary Fig. S2

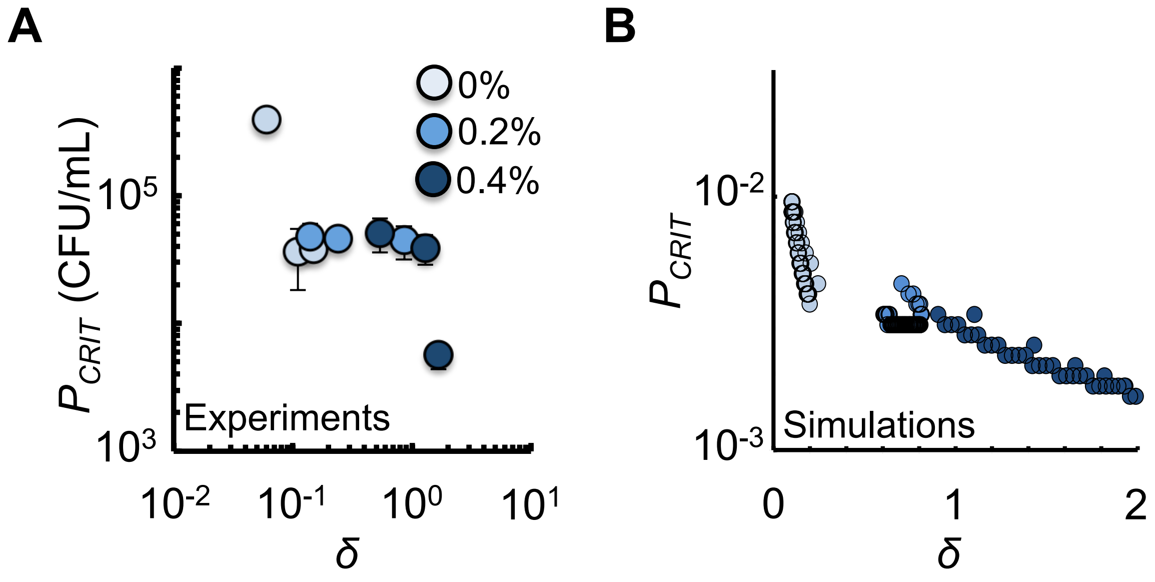

Supplementary Fig. S3

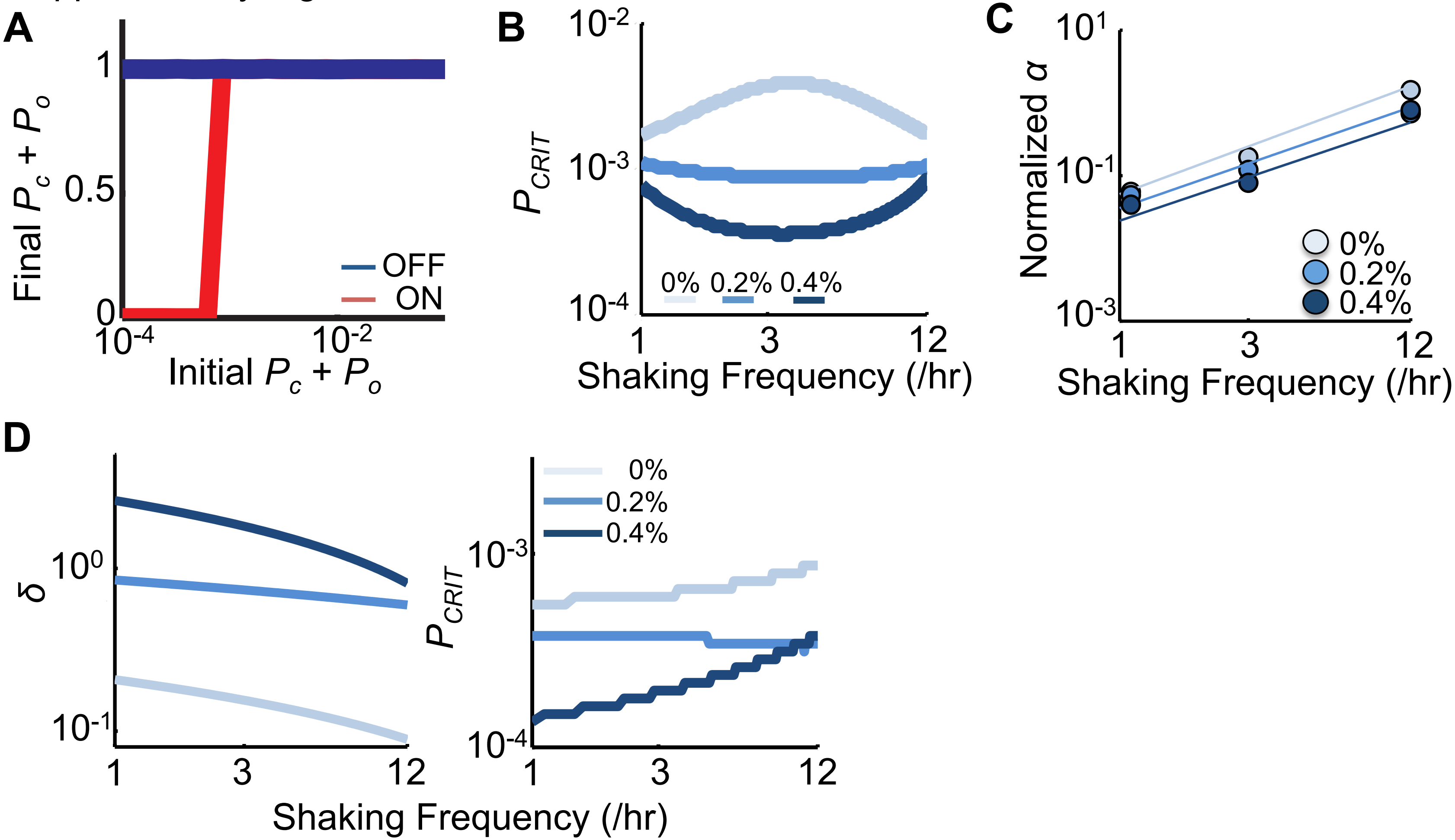

Supplementary Fig. S4

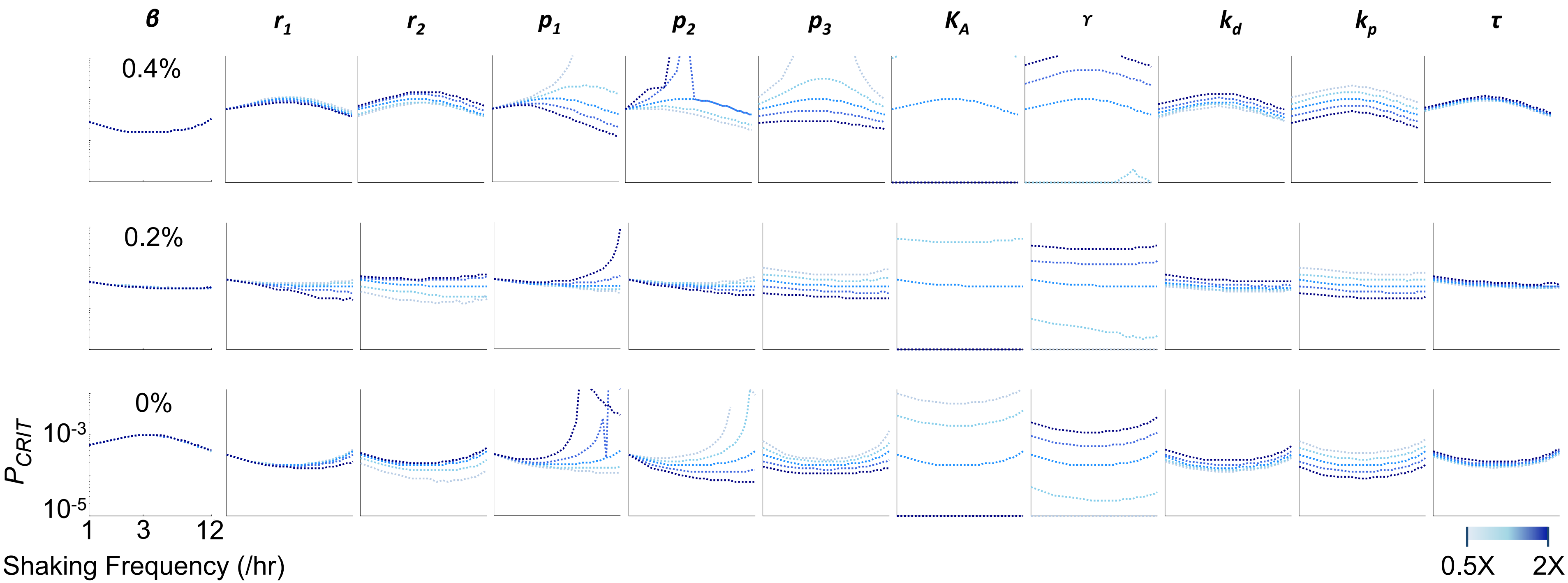

Supplementary Fig. S5

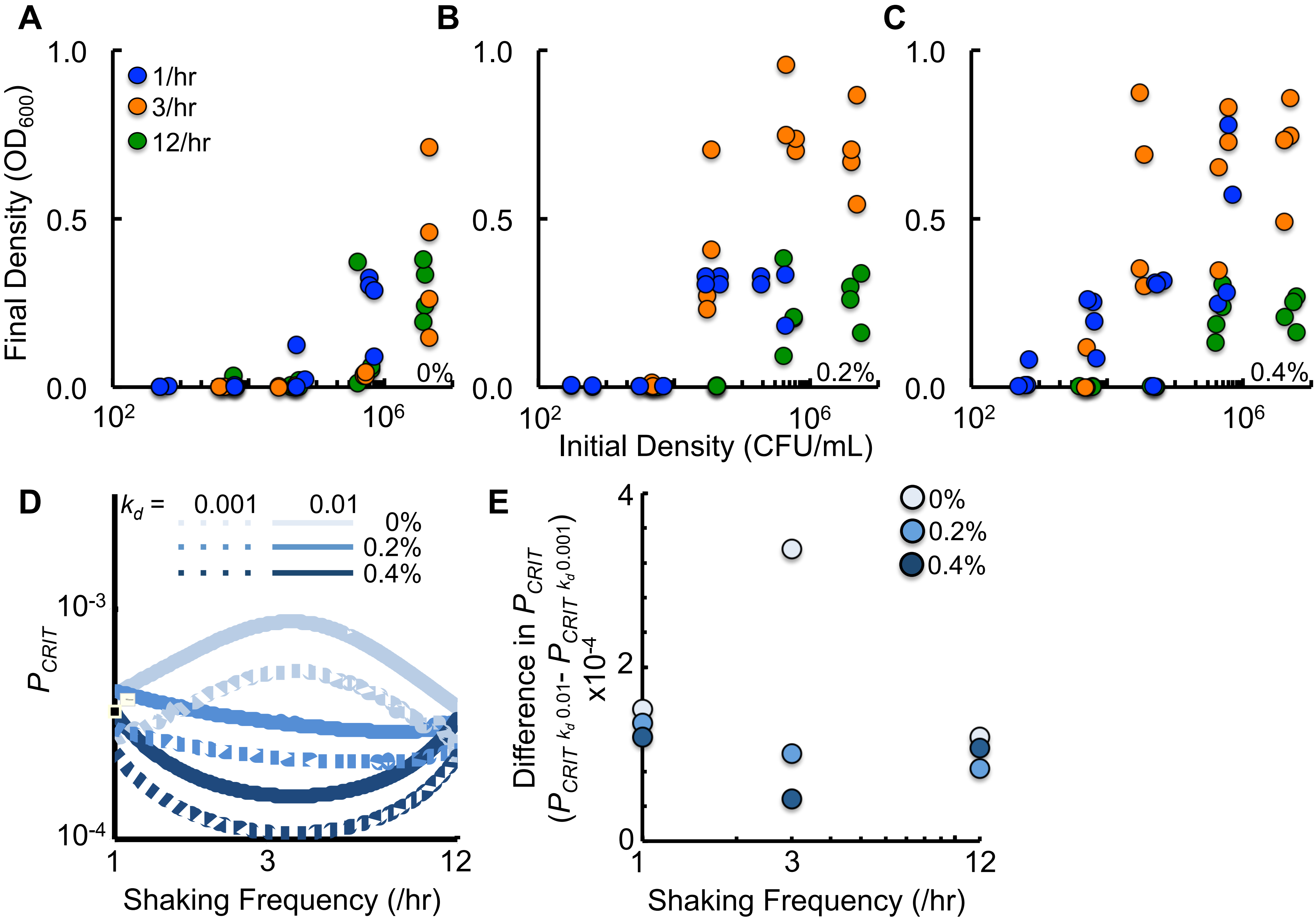

Supplementary Fig. S6

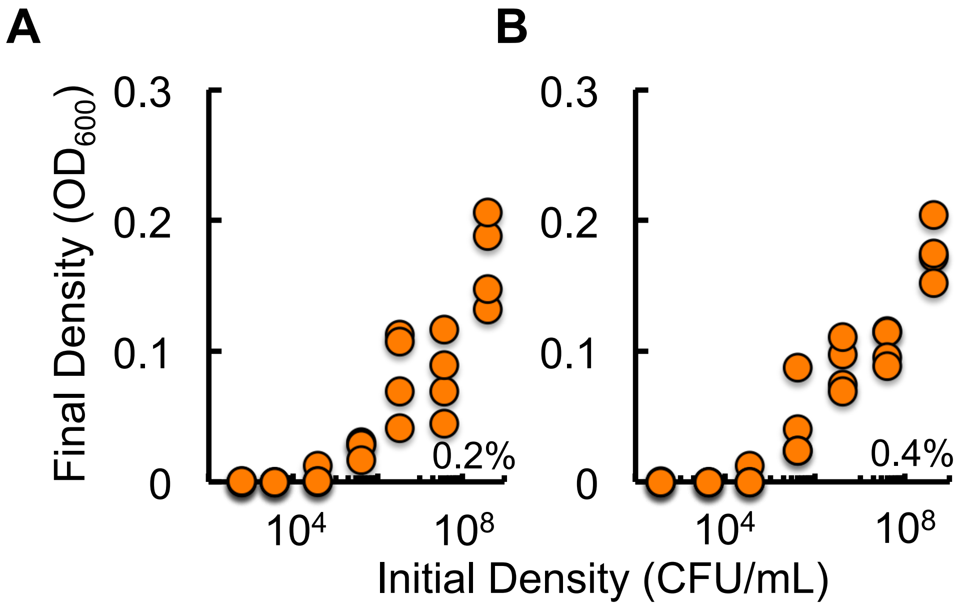

Supplement: Supplementary file 1 — Supplementary information [file 41598_2017_588_MOESM1_ESM.pdf]
